# Protective Efficacy of Cross-Reactive CD8^+^ T Cells Recognising Mutant Viral Epitopes Depends on Peptide-MHC-I Structural Interactions and T Cell Activation Threshold

**DOI:** 10.1371/journal.ppat.1001039

**Published:** 2010-08-12

**Authors:** Sophie A. Valkenburg, Stephanie Gras, Carole Guillonneau, Nicole L. La Gruta, Paul G. Thomas, Anthony W. Purcell, Jamie Rossjohn, Peter C. Doherty, Stephen J. Turner, Katherine Kedzierska

**Affiliations:** 1 Department of Microbiology and Immunology, University of Melbourne, Parkville, Australia; 2 The Protein Crystallography Unit, Department of Biochemistry and Molecular Biology, Monash University, Clayton, Australia; 3 Department of Immunology, St Jude Children's Research Hospital, Memphis, Tennessee, United States of America; 4 Department of Biochemistry and Molecular Biology, the Bio21 Molecular Science and Biotechnology Institute, University of Melbourne, Parkville, Australia; NIH/NIAID, United States of America

## Abstract

Emergence of a new influenza strain leads to a rapid global spread of the virus due to minimal antibody immunity. Pre-existing CD8^+^ T-cell immunity directed towards conserved internal viral regions can greatly ameliorate the disease. However, mutational escape within the T cell epitopes is a substantial issue for virus control and vaccine design. Although mutations can result in a loss of T cell recognition, some variants generate cross-reactive T cell responses. In this study, we used reverse genetics to modify the influenza NP_336–374_ peptide at a partially-solvent exposed residue (N->A, NPN3A mutation) to assess the availability, effectiveness and mechanism underlying influenza-specific cross-reactive T cell responses. The engineered virus induced a diminished CD8^+^ T cell response and selected a narrowed T cell receptor (TCR) repertoire within two Vβ regions (Vβ8.3 and Vβ9). This can be partially explained by the H-2D^b^NPN3A structure that showed a loss of several contacts between the NPN3A peptide and H-2D^b^, including a contact with His155, a position known to play an important role in mediating TCR-pMHC-I interactions. Despite these differences, common cross-reactive TCRs were detected in both the naïve and immune NPN3A-specific TCR repertoires. However, while the NPN3A epitope primes memory T-cells that give an equivalent recall response to the mutant or wild-type (wt) virus, both are markedly lower than wt->wt challenge. Such decreased CD8^+^ responses elicited after heterologous challenge resulted in delayed viral clearance from the infected lung. Furthermore, mice first exposed to the wt virus give a poor, low avidity response following secondary infection with the mutant. Thus, the protective efficacy of cross-reactive CD8^+^ T cells recognising mutant viral epitopes depend on peptide-MHC-I structural interactions and functional avidity. Our study does not support vaccine strategies that include immunization against commonly selected *cross-reactive* variants with mutations at partially-solvent exposed residues that have characteristics comparable to NPN3A.

## Author Summary

Introduction of a new influenza strain into human circulation leads to a rapid global spread of the virus due to minimal antibody immunity. Established T-cell immunity towards conserved viral regions provides some protection against influenza and promotes rapid recovery. However, influenza viruses mutate to escape the protective immunity. We found that established T cell immunity can recognise influenza mutants with variations at positions that are partially involved in T cell recognition. However, an initial priming with the mutated variant decreases recognition of the original parental virus. This finding results from a markedly lower functional quality and limited structural interactions of the mutant. In terms of possible vaccination strategies for rapidly changing viruses or tumours, it appears that priming with cross-reactive mutants that display such characteristics would be of no benefit as the same level of T cell immunity against such mutants can be elicited by exposure to the original virus.

## Introduction

Virus-specific CD8^+^ T cells play a critical role in host defence via the production of antiviral cytokines, the direct killing of virus-infected cells and the establishment of immunological memory [1]. The selection of CD8^+^ T cells into an immune response requires specific interaction between the T cell receptor (TCR) and virus peptide bound to Major Histocompatibility Complex class I (pMHC-I) molecules on the surface of infected host cells. The processing of virus proteins into short fragments generates thousands of peptides that might potentially form pMHC-I epitopes, but only a few elicit CTL responses [2].

Virus escape mutants are well documented for persistent infections and constitute a major problem for CD8^+^ T cell-mediated control and vaccine design [3], [4], [5], [6], [7], [8], [9]. With regard to the influenza A viruses, mutational changes driven by CD8^+^ cytotoxic T lymphocytes (CTLs) are unlikely to result in long-term persistence within the individual, as other mechanisms (particularly antibody) can ultimately mediate virus clearance [10]. Even so, the fact that such mutants can be found in nature suggests that influenza virus-specific CTLs are of protective value. Perhaps this reflects that the infection of new subjects favours the selection of mutant viruses that are more slowly controlled (and thus shed for longer), particularly in the face of a seasonal “bottleneck” where much of the population is already immune [11]. In humans, influenza escape variants have been observed for CD8^+^ T cell epitopes presented in context of several HLAs, including HLA-B8, HLA-B27 and HLA-B35 [12], [13], [14], [15], [16], [17], [18], [19]. The immunogenic peptides can be modified at an MHC anchor residue, resulting in defective binding to the MHC-I glycoprotein, or at a TCR contact site. Mutations at TCR contact residues lead to partial (cross-reactive) or total (non-cross-reactive) loss of recognition by wt CD8^+^ T cells [13], with some variants eliciting epitope-specific CD8^+^ T cell responses that are both novel and of substantial magnitude [12].

Using influenza A virus infection of B6 mice [20], we showed previously that virus variants with mutations at critical solvent-exposed residues that are important for TCR binding can generate effective but non-cross-reactive CD8^+^ CTL responses to what are essentially new epitopes [21], [22]. This raises the possibility that it might be worthwhile to think in terms of vaccinating against likely virus escape mutants. The present analysis focuses on the cross-reactive (to wt D^b^NP_366_) CD8^+^ T cell response to the mutant D^b^NPN3A epitope [22] formed by substitution of the partially solvent exposed, and non-prominent for TCR binding, residue at position (P) 3 of the influenza NP_366–374_ peptide [21], [23]. The findings have implications for vaccines to combat virus mutants and tumour escape variants, and suggest that immunisation against likely cross-reactive variants would have to be carefully evaluated to see if the strategy is worthwhile.

## Results

**Figure 1:**
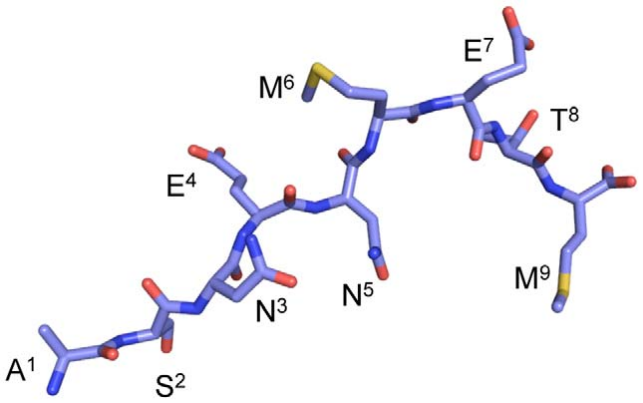
A stick conformation illustrating how the influenza NP_366–374_ interacts with H-2D^b^. NP_366–374_ peptide binds to the H-2D^b^ in an extended conformation. The P3-Asp, P5-Asn and P9-Met are the anchor residues, whereas the P4-Glu, P6-Met, P7-Glu and P8-Thr are solvent exposed and available for contact by the TCR.

Earlier analysis [21], [22] using sequential alanine substitutions in the immunogenic NP_366–374_ peptide established that there is some cross-reactive, though diminished, stimulation following the incubation of wt D^b^NP_366_-specific CD8^+^ T cells with the NP mutant peptide containing a single asparagine to alanine substitution at position (P) 3 (NPN3A mutant). Such cross-reactive CD8^+^ T cell responses after NPN3A stimulation are not surprising as the partially solvent exposed P3N residue is unlikely to play any prominent role in contacting the TCR [23]. The wt NP_366–374_ peptide binds to H-2D^b^ in an extended conformation where the P2-Ser, P5-Asn and P9-Met are the anchor residues, P3-Asp is a semi-anchor (partially buried/partially exposed), and P4-Glu, P6-Met, P7-Glu and P8-Thr are solvent exposed and available for contact by the TCR (Fig. 1). This provides the basis for a defined experimental system that can be used to determine what happens when a TCR repertoire is selected to what would seem (from *in vitro* analysis) to be a suboptimal, cross-reactive mutant epitope. The interpretation of *in vitro* analysis alone should, however, be tempered with caution, as another mutant (M6A) that did not cross-react at all with the wt epitope elicited a completely novel, *in vivo* endogenous CTL response of equivalent magnitude when expressed in an infectious influenza A virus [22]. What would be the case for TCR responses elicited by influenza A viruses expressing the mutant NPN3A peptide in the native viral protein?

### Consequences of 1^o^ and 2^o^ homologous challenge for CTL response magnitude

**Figure 2:**
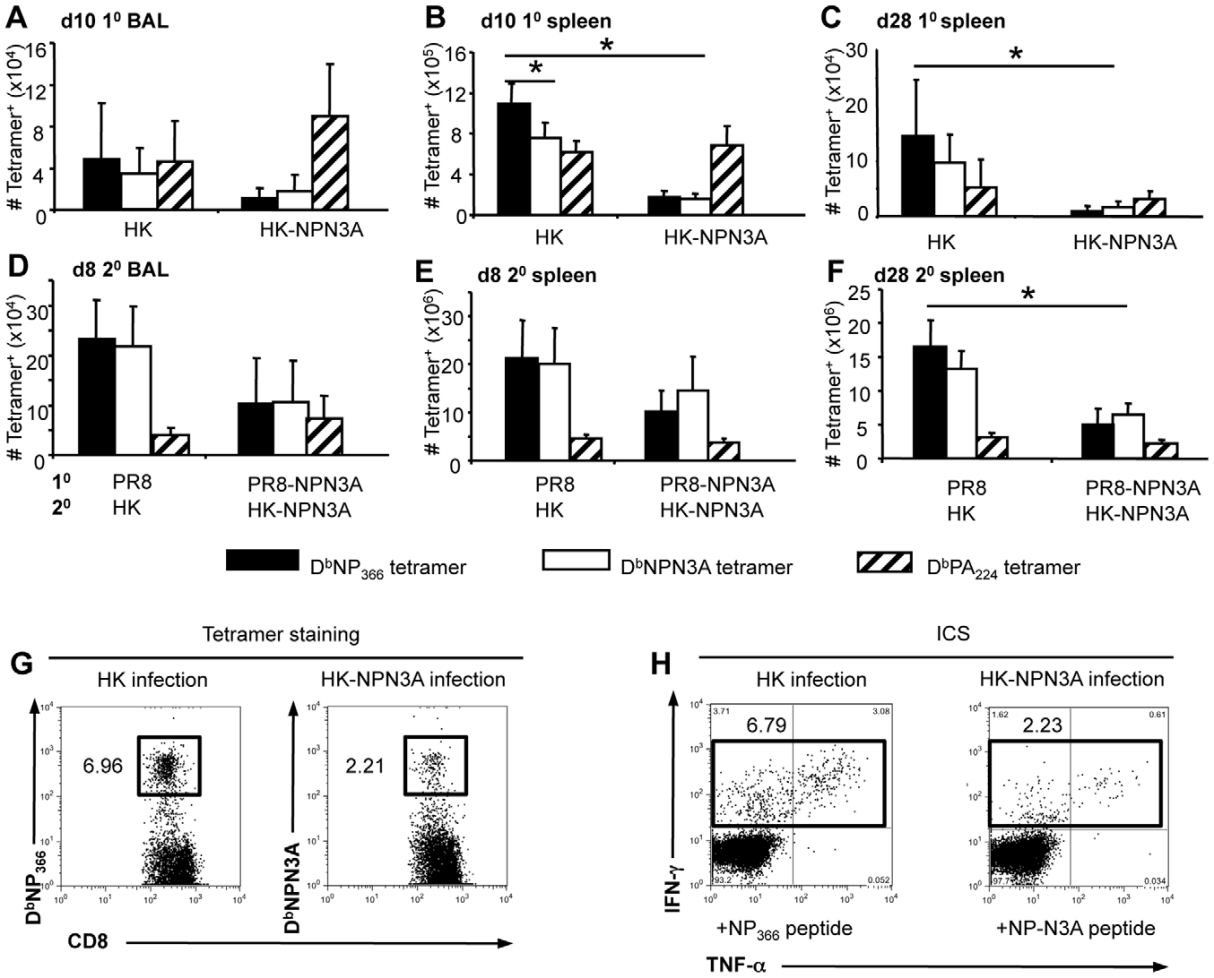
Acute and recall CD8^+^ T cell responses to D^b^N3A and D^b^NP_366_. The magnitude of CD8^+^ T cell responses at the peak (d10 1^o^ A,B; d8 2^o^ D,E) or memory (d28, CF) phases following 1^o^ (A–C) or 2^o^ (D–F) infection. Cells were stained with the D^b^NP_366_-PE, D^b^NPN3A-PE or D^b^PA_224_-PE tetramers and anti-CD8-PerCPCy5.5 mAb. The numbers of epitope specific CD8^+^ T cells were calculated from the % cells staining and the total cell counts. The wt HK or HK-NPN3A viruses were used for 1^o^ i.n. infection and 2^o^ i.n. challenge of i.p.-primed (PR8 or PR8-NPN3A i.p. >30d previously) B6 mice. Data are mean±SD n = 5 mice per group, * = p<0.01. Memory T cell numbers were also analysed on d60, and the tetramer analysis was replicated using the ICS assay (data not shown). (G, H) Representative dot plots are shown for (G) D^b^NP_366_ or NPN3A tetramer staining and (H) intracellular cytokine assay after infection with either HK or HK-NPN3A for the same individual mouse.

The NPN3A mutation was engineered into PR8 (H1N1) and HKx31 (H3N2) influenza viruses (PR8NPN3A, HKNPN3A) to allow cross-challenge experiments in the absence of antibody neutralisation. The B6 mice were immunised i.p. with the virulent PR8 mutant and wt viruses, while the HK viruses were used for primary (1^o^) i.n. infection of naïve mice or secondary (2^o^) i.n. challenge of PR8-immune (>30d previously) mice. Naïve (primary) or PR8-immune (PR8, or PR8NPN3A) mice were challenged i.n. with the homologous virus (HK, or HKNPN3A) and CD8^+^ T cell responses were measured using the D^b^NP_366_ and D^b^NPN3A tetramers (Fig 2). It was immediately apparent that the splenic D^b^NPN3A^+^CD8^+^ set elicited by the HKNPN3A challenge was significantly smaller on d10 (p<0.05) than the D^b^NP_366_^+^CD8^+^ T cell response induced by the wt virus (Fig 2B), though there was no significant difference between D^b^NPN3A^+^CD8^+^ and D^b^NP_366_^+^CD8^+^ T cell numbers at the site of infection (BAL, Fig 2A). This has been seen before [24] and suggests that the need to clear virus from the lung results in preferential CTL localization to the site of infection when immune T cell numbers are limited. The profile of a diminished D^b^NPN3A^+^CD8^+^ T cell response was maintained into memory (d28, Fig 1C; p<0.02), supporting the view that the relative size of persistent T cell pools reflects the extent of antigen driven proliferation during the acute anti-viral response.

A high proportion of the wt D^b^NP_366_^+^CD8^+^ T cells in BAL (94.0% (1^0^, d10); 93.2 (2^0^, d8); Fig 2A) and spleen (69.8% (1^0^, d10); 93.7% (2^0^, d8); Fig 2B) bound the D^b^NPN3A tetramer and produced IFN-γ when stimulated by the NPN3A peptide (data not shown). Similarly, the majority (≥90%) of CD8^+^ T cells elicited by HKNPN3A infection bound the D^b^NP_366_ tetramer and produced IFN-γ (data not shown) at levels equivalent to those seen for the response to NPN3A, indicating a high level of cross-reactivity between the D^b^NP_366_^+^CD8^+^ and NPN3A^+^CD8^+^ T cell responses. Staining with the D^b^PA_224_ tetramer was included to establish that the NPN3A mutation neither diminished nor enhanced other influenza-specific CD8^+^ T cell responses (Fig 2).

Despite the decreased D^b^NPN3A^+^CD8^+^ T cell numbers generated following primary infection (Fig 2A–C), the recall response was substantial following HKNPN3A challenge of PRNPN3A-immune mice (Fig 2DE) and the NP>PA immunodominance hierarchy that has long been recognized for secondary responses to wt influenza A viruses in H2^b^ mice [25] was maintained (Fig 2DE). Similarly, the total cell numbers for memory D^b^PA_224_-specific T cells on d28 were comparable for those primed and boosted within the wt and mutant virus combinations (Fig 2F), while the D^b^NPN3A^+^ CD8^+^ set was 8-fold smaller than the wt D^b^NP_366_^+^CD8^+^ population (p<0.005). Again, the results following secondary challenge support the view that, at least in the earlier (d28) stages of memory, T cell numbers reflect the extent of clonal expansion during the acute phase [26]. In our experiments, we detected D^b^NP_366_^+^CD8^+^ and NPN3A^+^CD8^+^ T cells by two techniques, tetramer staining and IFN-γ ICS. Both techniques gave us comparable antigen-specific CD8^+^ T cell numbers, indicating that both tetramers accurately detected epitope-specific populations (Fig. 2GH).

Given that the NPN3A mutation was associated with a numerically diminished response following infection with either the wt or NPN3A influenza A viruses (Fig 2), we also asked if there was any effect on CD8^+^ T cell function or phenotype, particularly for markers (CD62L and CD127) that discriminate between memory T cell subsets [27], [28]. More of the D^b^NPN3A^+^CD8^+^ T cells remained CD62L^hi^ when sampled at the peak of the response (d10) following primary challenge (Fig S1A), confirming the impression from the quantitative analysis (Fig 2 A–C) that there may be less clonal expansion. On the other hand, IL-7R expression was comparable for the D^b^NPN3A^+^CD8^+^ and D^b^NP_366_^+^CD8^+^ T cell populations generated in virus-infected naïve mice (Fig S1 CD) with both being (p<0.01) lower than the values for the D^b^PA_224_-specific set (Fig S1C). This suggests that IL-7R levels may be antigen dose- rather than magnitude-dependent. Neither of these differential effects was apparent for d28 memory T cells specific for the mutant or wt epitopes (Fig S1BD). Functional analysis of cytokine production based on short term (5h) stimulation with cognate peptide in the ICS assay showed no obvious differences at any stage for the D^b^NP_366_ and D^b^NPN3A-specifc T cells, though the usual divergence [29] from the D^b^PA_224_^+^CD8^+^ T cell response was observed (p<0.01) (Fig S1 E–H). These data suggest that NPN3A mutation leads to cross-reactive, but diminished, CD8^+^ T cell responses with comparable cytokine production profiles.

### Crystal structure and thermostability of the H2Db-NP-N3A complex

**Figure 3:**
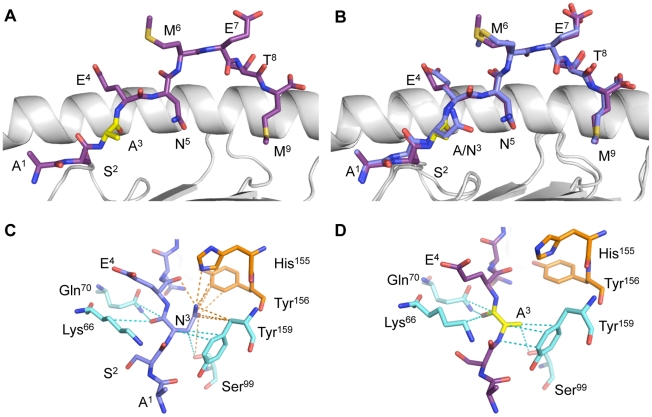
Structure of the D^b^NP_366–374_ and D^b^NPN3A complexes. The H2D^b^ molecule is in a cartoon representation with the α1-helix on the back and the α2-helix removed for better clarity. The peptide is represented in stick conformation with the C terminus on the right. (A) The NPN3A is in purple and (B) the NP_366_ in blue, with the p3 mutation in yellow. (B) Overlay of the peptide-binding cleft for H2D^b^NP_366_ and H2D^b^NPN3A with the α1-helix on top and the α2-helix on the bottom. (C) Contacts with the H-2D^b^ molecule by (C) P3N in NP_366_ and (D) P3A in NPN3A. The Asn3 mutation to alanine abolishes contacts between the P3 residue of the peptide with His155 and the Tyr156 of the H-2D^b^.

Can the smaller response to D^b^NPN3A be correlated with structural constraints or any decrease in stability for the pMHC-I complex? The D^b^NPN3A crystal structure containing the heavy chain of H-2D^b^ (residues 1–275), the β2 microglobulin (residues 1–99) and the 9 residues of the NPN3A peptide was determined to a 2.6 Å resolution (Fig. 3 and Table 1), with a final R_factor_ of 22.1% and an R_free_ of 30.4%. The structure of the H-2D^b^-NPN3A complex was compared (Fig. 3B, Table 1) to the wt D^b^NP_366_ [23]. As observed for the wt D^b^NP_366_, the mutant NPN3A peptide bound H-2D^b^ in an extended conformation, the P2-Ser, P5-Asn and P9-Met represent the anchor residues, P3-Ala semi-anchor residue, whereas the P4-Glu, P6-Met, P7-Glu and P8-Thr are solvent exposed and available for contact by the TCR (Fig. 3AB). The D^b^NP_366_ and D^b^NPN3A structures are very similar with a root mean square deviation (r.m.s.d.) of 0.44 Å on the α1–α2 domains and a r.m.s.d of 0.20 Å on the peptides. With the exception of P3-Ala, the structure of D^b^-NP_366_ and D^b^-NPN3A superimpose well.

**Table 1:** Data collection and refinement statistics.

Data Collection Statistics	H2D^b^-NPN3A
Temperature	100K
Space group	*I222*
Cell Dimensions (a,b,c) (Å)	93.64, 94.98, 132.24
Resolution (Å)	50.00 - 2.60 (2.70-2.60)
Total number of observations	131799 (13218)
Number of unique observations	18241 (1905)
Multiplicity	7.2 (6.9)
Data completeness (%)	98.6 (97.7)
I/σ_I_	27.55 (6.37)
R_merge_ a (%)	6 (30.3)

Refinement Statistics	
Non-hydrogen atoms	
Protein	3040
Water	46
Resolution (Å)	2.60
*R_factor_* b (%)	22.1
*R_free_* b (%)	30.4
Rms deviations from ideality	
Bond lengths (Å)	0.012
Bond angles (°)	1.468
Ramachandran plot (%)	
Most Favoured Region	85.5
Allowed Region	12.5
Generously allowed region	1.7
B-factors (Å^2^)	
Average main chain	40.806
Average side chain	40.807

a R_merge_ = Σ|I_hkl_−<I_hkl_>|/ΣI_hkl_.

b R_factor_ = Σ_hkl_| |F_o_|−|F_c_| |/Σ_hkl_|F_o_| for all data except ≈5% which were used for R_free_ calculation. Values in parentheses are for the bin of highest resolution (approximate interval = 0.5 Å).

However, the mutation of P3-Asn to Ala leads to a loss of 35 contacts between the peptide and the MHC molecule. In comparison to the wt NP_366_ that makes 636 contacts with the H-2D^b^ molecule, the NPN3A peptide achieves only 601 contacts. Interestingly, the Asn3 Ala mutation abolishes contacts between the P3 residue of the peptide with His155 and the Tyr156 of the MHC and eliminates one hydrogen bond between the P3-Asn^Nδ2^ and the P4-Glu^O^ (Fig. 3CD). The loss of inter-residue bonding between 3N and 4E within the NPN3A peptide may also be important for TCR recognition as it changes the characteristics of the wt D^b^NP_366_ epitope (defined as type III constraint) [30] into an unconstrained D^b^NPN3A epitope. This change of constraint within the epitope could affect the dynamics of the peptide upon TCR ligation, and subsequently alter TCR reactivity toward the mutated epitope. In addition, the loss of contacts with the His155 of the MHC is of interest, as position 155, termed the “gate-keeper residue” [31] is involved in contacting the TCRs in all TCRpMHC-I complexes solved to date [32]. Thus, the lack of interactions between the NPN3A peptide and the MHC through His155 may also affect recognition of the complex by D^b^NP_366_-specific TCRs.

The loss of contacts between the peptide and the MHC molecule could lead to decreased stability of the peptide and subsequent changes in NPN3A presentation. To test this hypothesis, we performed a thermostability assay on both NP_366_ and NPN3A bound to the H-2D^b^ molecule. The NP_366_ and NPN3A peptides are equally effective at stabilising H-2D^b^. The pMHC-I complex with the NP_366_ wt peptide had a Tm of 51.8±0.7°C and D^b^NPN3A showed a comparable level of thermostability (Tm = 51.4±1°C), irrespective of the concentrations of the complex used for the assay. This suggests that the NPN3A mutation does not modify the stability of the pMHC-I complex when compared to the cognate epitope.

### Naïve precursor frequency and TCR repertoire for D^b^NPN3A

**Figure 4:**
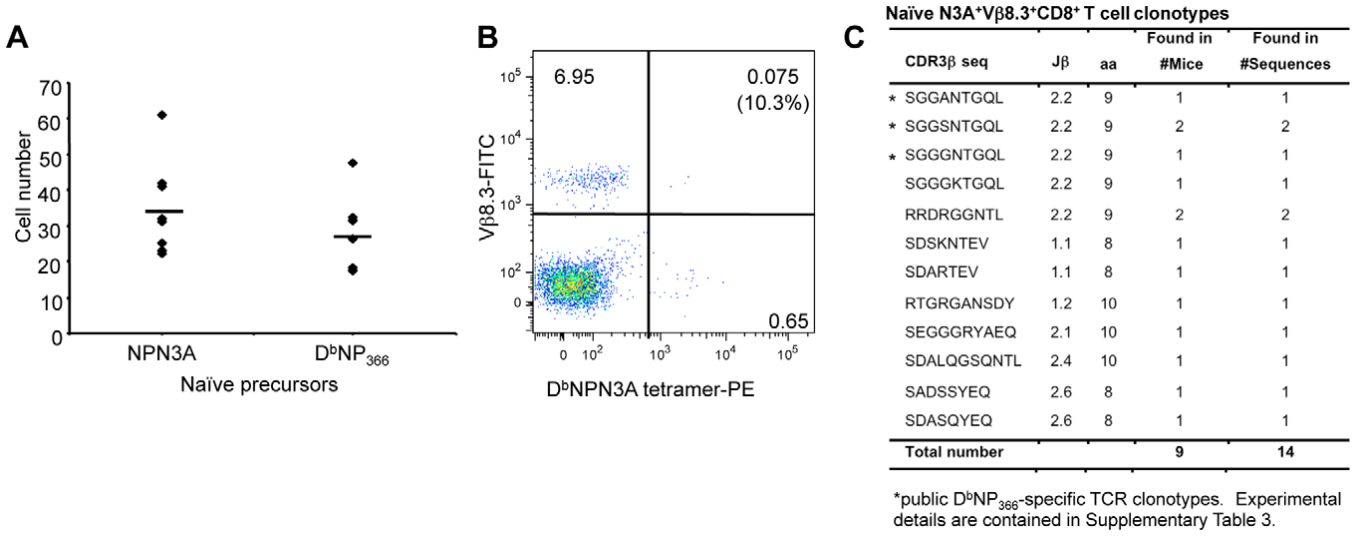
TCR repertoire of naïve D^b^NPN3A^+^CD8^+^ T cells. Effects of the N3A substitution within NP_366–374_ on (A) naïve T cell precursor frequency, (B) Vβ8.3 bias and (C) naïve TCRβ repertoire within the D^b^NPN3A^+^Vβ8.3^+^CD8^+^ set. (A) Naïve D^b^NPN3A^+^ and D^b^NP_366_^+^CD8^+^ T cell precursors within lymph nodes and spleens were identified with the D^b^NPN3A-PE and D^b^NP_366_-PE tetramers using an established tetramer enrichment protocol [53]. (B) Naïve D^b^NPN3A^+^CD8^+^ T cells were assessed for the conservancy of Vβ8.3 bias. (C) a CDR3β sequences within the naïve D^b^NPN3A^+^Vβ8.3^+^CD8^+^ T cells were determined by a single-cell PCR, amplification of Vβ8.3 transcripts and sequencing.

Is the smaller D^b^NPN3A^+^CD8^+^ T cell response a consequence of diminished naïve CTL [33]? We found (Fig 4A) similar naive CTLp counts for D^b^NPN3A (34.6±13.08) and D^b^NP_366_ (28.5±11.0), indicating that the smaller response to D^b^NPN3A^+^CD8^+^ is not due to reduced number of naïve precursors. Furthermore, assessing the extent of Vβ8.3 bias (the dominant Vβ for the D^b^NP_366_^+^CD8^+^ set) within the naïve D^b^NPN3A^+^CD8^+^ population (Fig 4B) showed that the extent of Vβ8.3 usage (mean 13.2%±4.3) (Fig. 4B) was much the same as that determined previously [33] for naïve D^b^NP_366_^+^CD8^+^ CTLps (mean 17.1%±7.4) However, sequencing the naïve D^b^NPN3A^+^Vβ8.3^+^CD8^+^ TCR CDR3β regions showed a clear difference from the comparable wt-specific set. The “public TCR” dominance characteristic of D^b^NP_366_^+^Vβ8.3^+^CD8^+^ T cells in both pre-immune [33] and immune [34] TCRβ repertoires [34] was not a prominent feature of the D^b^NPN3A-specific TCR repertoire. These public TCRs were found in only one (SGGANTGQL and SGGGNTGQL) or two (SGGSNTGQL) of the 10 mice tested (Fig. 4C). Thus, although the naïve CTLp frequencies are comparable for D^b^NP_366_^+^Vβ8.3^+^CD8^+^ and D^b^NPN3A^+^Vβ8.3^+^CD8^+^ T cells and there is some overlap of some cross-reactive TCRs, the two repertoires are far from identical. The roughly equivalent numbers of precursors specific for the wt D^b^NP_366_ and mutant NPN3A peptides were unexpected considering the lower response after infection with the mutated virus. The lower magnitude of the NPN3A^+^CD8^+^ T cell response and narrower TCRβ repertoire suggest that only a proportion of naïve NPN3A^+^CD8^+^ precursors are being recruited into the immune response or that, once recruited, these cells do not expand efficiently. Inefficient recruitment and/or expansion early after influenza infection, despite large naïve CTL precursor numbers, have been recently documented by our group as key determinants of subdominance for D^b^PB1-F2_62_ and K^b^NS2_114_-specific responses [33].

### D^b^NPN3A selects distinct and more restricted TCR Vβ signatures

The next step was to dissect the immune D^b^NPN3A^+^CD8^+^ CTL repertoire to determine how TCR diversity relates to the size of the immune D^b^NPN3A^+^CD8^+^ T cell response. The D^b^NPN3A-specific CD8^+^ T cells were first analysed for Vβ usage by staining with a panel of anti-TCRVβ mAbs and the D^b^NPN3A tetramer. After infection with the NPN3A viruses, the strong Vβ8.3 bias characteristic of responding D^b^NP_366_^+^CD8^+^ T cells [34], [35] was prominent for the D^b^NPN3A^+^CD8^+^ sets in only 50% of the immune mice (n = 10). The mutant D^b^NPN3A^+^CD8^+^ T cells utilized a variable spectrum of TCRVβ elements, with over-representation of Vβ 7, 8.1/8.2, 9, 11, and 12 after primary (Fig S2A) or Vβ6, 7 and 9 following secondary (Fig S2B) NPN3A virus challenge.

We analysed TCRβ clonotypes within the Vβ8.3 as (i) it is still a preferred region (29.8%±20.2) of NPN3A^+^CD8^+^ T cell response) and (ii) clonotypes within this region could be highly relevant for cross-reactive CD8^+^ T cell responses between NP_366_ and NPN3A as they are prominent in both populations. Overall, the mutant and the wt immune Vβ8.3 repertoires appear different. Single-cell RT-PCR and sequencing of the CDR3β region of tetramer^+^Vβ8.3^+^CD8^+^ T cells following either HK or HK-NPN3A infections showed that (Table S1) the mutant D^b^NPN3A^+^CD8^+^ T cells utilized different Jβ regions (primary (1^0^): Jβ1.1, Jβ1.3; secondary (2^0^): Jβ1.1, Jβ1.6, Jβ2.2) in comparison to the wt D^b^NP_366_^+^CD8^+^ population (1^0^: Jβ2.2, Jβ2.4; 2^0^: Jβ 2.2), and showed evidence of more variable CDR3β loop lengths (8–9 aa). The reduction (relative to the wt D^b^NP_366_) in Vβ8.3 usage by the D^b^NPN3A^+^CD8^+^ T cells reproduces the relative loss of “public” wt TCRs found for the naïve repertoire (Fig 4B). This likely reflects that the N3A mutation has disrupted an “optimal” TCR/pMHC fit that maximizes antigen-driven clonal expansion. Indeed, the public TCRs were not a prominent feature of the D^b^NPN3A^+^CD8^+^ set, being found in only 4 of the 7 NPN3A-infected mice at a very low frequency, namely 0% in the 1^0^ response and 22.9% in the 2^0^ response (Table 2). This is in contrast to the D^b^NP_366_^+^CD8^+^ Vβ8.3^+^ TCR repertoire [34] that is largely (90%) comprised of high-frequency, public clonotypes found in all infected B6 mice [34].

**Table 2:** Frequency of TCRβ clonotypes in D^b^NPN3A^+^ Vβ8.3^+^CD8^+^ T cells after 1^0^ (M1, M2) and 2^0^ (M3 to M7) mutant HK-NPN3A infection detected with either the D^b^NP_366_^+^ or D^b^NPN3A^+^ tetramer.

HK-NPN3A infection			1^0^ response				2^0^ response									
			M1		M2		M3		M4		M5		M6		M7	
CDR3β	Jβ	aa	NP	N3A	NP	N3A	NP	N3A	NP	N3A	NP	N3A	NP	N3A	NP	N3A
SGGANTGQL	2S2	9							3			9		2		5
SGGSNTGQL	2S2	9										11	1		2	3
SGGGSTGQF	2S2	9					11									
SGGGNTGQL	2S2	9							12	1.5			99	3		
SGGGNNGHL	2S2	9					2									
KGGGNTGQL	2S2	9									100	80				
SDAASTEV	1S1	8			84	88										
SDAANTEV	1S1	8			16	12			19	11						
SDAVATEV	1S1	8							62	82						
SDASSTEV	1S1	8													98	92
SDSANTEV	1S1	8							3	4.5						
RRDRGGNTL	1S3	9	95	100												
SGGTENSPL	1S6	9												95		
SDAQLYAEQ	2S1	9	2													
SVGGRAEQ	2S1	8								1.5						
SDWGSQNTL	2S4	9					87	100								
SDGGGTYEQ	2S5	9	2													
**TOTAL sequences**			**44**	**42**	**32**	**43**	**64**	**32**	**34**	**66**	**59**	**35**	**72**	**61**	**50**	**61**

M: individual mouse; NP: D^b^NP_366_ tetramer; N3A: D^b^NPN3A tetramer.

1^0^ responses were generated by i.n. HK-NPN3A infection of mice; 2^0^ responses were generated by priming mice with i.p. PR8-NPN3A virus then challenging with i.n. HK-NPN3A virus; D^b^NP_366_: complex of H2D^b^ and NP_366–374_ peptide; D^b^NPN3A: complex of H2D^b^ and NPN3A_366–374_ peptide.

These results also establish that the naïve D^b^NPN3A^+^ CD8^+^ TCR repertoire (Fig. 4C) is predictive of the immune D^b^NPN3A^+^ CD8^+^ TCRβ response (Table 2). The relative lack of a “public” response translated to a profile of reduced “sharing” between individual mice, and a total increase in the number of D^b^NPN3A^+^CD8^+^ clonotypes due to the ‘private’ nature of TCRβ repertoire for each mouse (Table 2). Even so, the clonotypic diversity within individual NPN3A virus-infected mice was reduced compared to the spectrum found following wt virus-infection. This was true whether the cells were sorted using the wt D^b^NP_366_ or the mutant D^b^NPN3A tetramer, reflecting the significant cross-reactivity between the D^b^NP_366_ and D^b^NPN3A-specific populations recovered from mice infected with the NPN3A viruses. By the measure of tetramer binding, it thus seems that the NPN3A virus is selecting a less diverse repertoire than the wt virus, with the repertoire being almost completely cross-reactive with that elicited by the wt D^b^NP_366_ epitope.

Similarly, when the D^b^NP_366_^+^Vβ8.3^+^CD8^+^ T cells induced by wt HK infection were sequenced, the majority of the TCRβ clonotypes were detected with both the D^b^NP_366_ and D^b^NPN3A tetramers (Table 3). However, a switch in frequency was seen for some CDR3β-defined clonotypes, indicating selective binding of particular TCRs by the D^b^NPN3A tetramer. For example in M9 and M10 (Table 3) the ‘public’ SGGANTGQL CDR3β-sequence dominated the D^b^NP_366_^+^Vβ8.3^+^CD8^+^ T cell population, whereas this sequence was less commonly detected in the same mice by the D^b^NPN3A tetramer. The difference could, of course, reflect diversity in TCRα usage.

**Table 3:** Frequency of TCRβ clonotypes in D^b^NP_366_^+^Vβ8.3^+^CD8^+^ T cells after 1^0^ (M8–M10) and 2^0^ (M11–M13) wt influenza virus infection detected with either the D^b^NP_366_^+^ or D^b^NPN3A^+^ tetramer.

HK infection			1^0^ response						2^0^ response					
			M8		M9		M10		M11		M12		M13	
CDR3β	Jβ	aa	NP	N3A	NP	N3A	NP	N3A	NP	N3A	NP	N3A	NP	N3A
SGGANTGQL	2S2	9	67	70	53	3	63	22	10	15	36	48	24	22
SGGSNTGQL	2S2	9	22	16	2	17	27		50	13	2		50	16
SGGGNTGQL	2S2	9	11	7										5
SGGGSTGQL	2S2	9				17	7							
RGGSNTGQL	2S2	9			2						4			
RGGANTGQL	2S2	9							5	13	15	36	6	11
RGGGNTGQL	2S2	9										4		
RGGAPTGQL	2S2	9							10					
KGGSNTGQL	2S2	9				63	3				38	2		
KGGGNTGQL	2S2	9							25	60	4	4		3
SGGQGNSPL	2S2	9										2		
KAGGSTGQL	2S2	9						48						
KAGGGTGQL	2S2	9		5										
RALGRNTEV	1S1	9			2			3						
SDAGKTEV	1S1	8											6	30
RDSANTEV	1S1	8										2	15	8
SDAGAEQ	2S1	7		2										5
SDWGWQNTL	2S4	9			40			27						
**TOTAL sequences**			**27**	**43**	**45**	**30**	**30**	**60**	**40**	**47**	**47**	**44**	**34**	**37**

M: individual mouse; NP: D^b^NP_366_ tetramer; N3A: D^b^NPN3A tetramer;

1^0^ responses were generated by i.n. HK infection of mice; 2^0^ responses were generated by priming mice with i.p. PR8 viruses then challenging i.n. with the HK virus; D^b^NP_366_: complex of H2D^b^ and NP_366–374_ peptide; D^b^NPN3A: complex of H2D^b^ and NPN3A_366–374_ peptide.

*Predominant: ≥15%, ^#^Common: present in all mice sampled.

Following clonotype selection with the D^b^NP_366_ and D^b^NPN3A tetramers, sequencing of the secondary TCRβ repertoire (M11–M13) induced by challenge with the homologous (PR8, then HK) viruses showed less divergence within the wt D^b^NP_366_^+^CD8^+^ T cell specific population. Interestingly, clonotypes like KGGSNTGQL were enriched by the D^b^NPN3A tetramer in some but not other mice (Table 3; present in M9 but not M12) suggesting, again, the likely importance of Vβ-Vα chain pairing for recognition of the native D^b^NP_366_^+^CD8^+^ T cells with the mutant D^b^NPN3A tetramer. Thus, the analysis suggests that only a subset of the repertoire generated by wt infection is able to recognize the D^b^NPN3A epitope, though this population is more diverse than that generated in response to the mutant NPN3A virus. All the statistical differences (Table 2, Table 3) were confirmed when the data were standardized to a number of sequences (data not shown).

We further analyzed the D^b^NPN3A^+^Vβ9^+^CD8^+^ set that was prominent in 2 of the HK-NPN3A secondarily challenged mice. An average of 1.5±1 TCRβ clonotypes was found within this population (Table S2). Again, the D^b^NPN3A^+^Vβ9^+^CD8^+^ TCRβ repertoire emerged as essentially restricted and private. However, TCRβ analysis within other Vβs for NPN3A^+^CD8^+^ T cells would need to be performed to compare the whole TCR repertoires.

Analysis of Vα chain usage for the mutant D^b^NPN3A^+^CD8^+^ and wt D^b^NP_366_^+^CD8^+^ T cells by PCR with a panel of Vα specific primers established that those two T cell responses indeed tend to utilise different Vα chains. The wt D^b^NP_366_^+^CD8^+^ T cell population [36] (Day EB, unpublished) tended to use Vα8 and Vα17.3 (n = 3). Conversely, the D^b^NPN3A^+^CD8^+^ TCRs preferentially expressed Vα4, Vα5 and Vα11 (Table S1). While the sample size is small and there are at least 72 different Vα chains, these results provide a snapshot of the mutant D^b^NPN3A^+^ and wt D^b^NP_366_^+^ populations and suggest that there are differences in both Vβ and Vα TCR chain usage.

### Response characteristics following heterologous challenge and TCR/pMHC-I avidity

**Figure 5:**
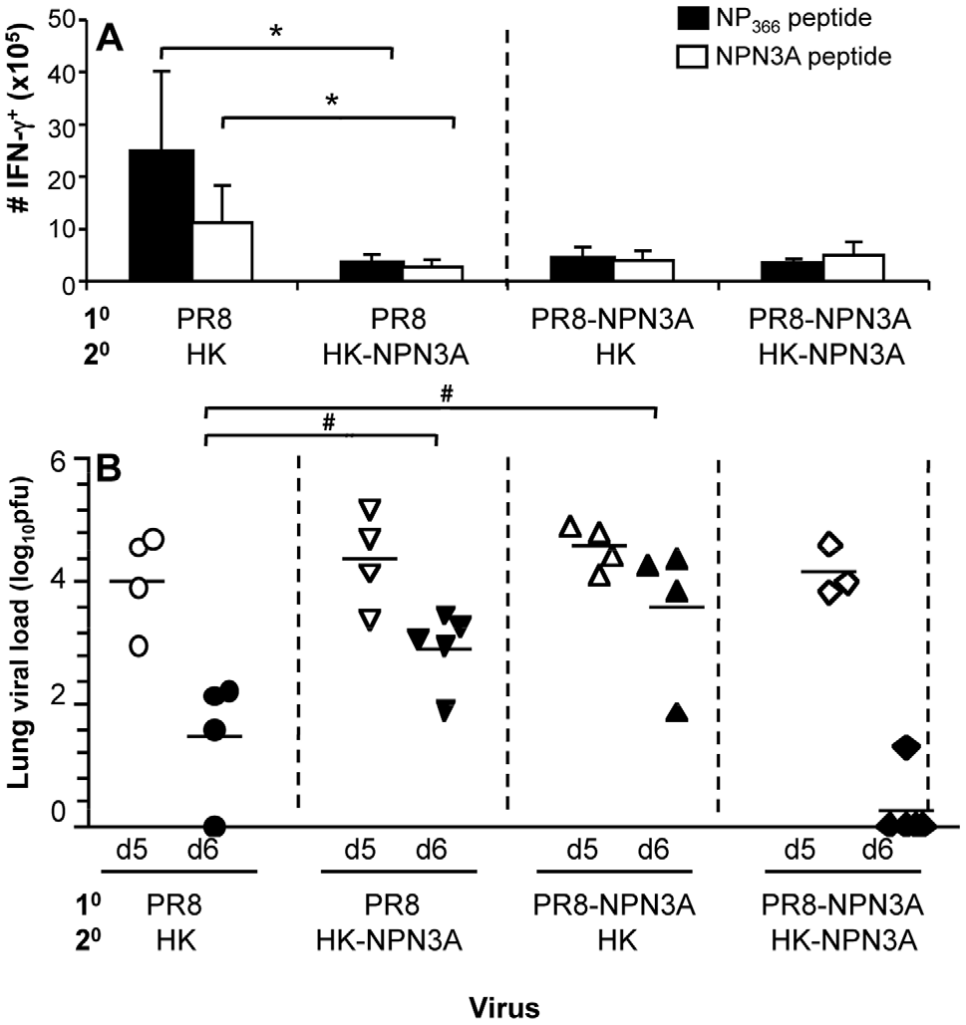
Heterologous 2^o^ challenge with the wt HK and mutant HK-NPN3A viruses. Naïve (A) B6 or (B) μMT mice were primed i.p with the mutant PR8-NPN3A and challenged i.n. 6 weeks later with 1×10^4^ p.f.u. of either the wt HK or the mutant HK-NPN3A virus. Alternatively, naïve mice were primed i.p. with the wt PR8 virus and challenged i.n. 6 weeks later with 1×10^4^ p.f.u. of the wt HK or the mutant HK-NPN3A virus. (A) The magnitude of the CD8^+^ T cell response measured 8d after 2^0^ infection was determined by the ICS assay for IFN-γ. Data represent mean±SD (n = 5) (B) Viral clearance after the secondary homologous (PR8->X31 and PR8-NP-N3A>X31-NPN3A) or heterologous (PR8->X31-NPN3A and PR8-NP-N3A>X31) challenge. Lungs were sampled at days 5 and 6 after secondary infection and homogenized for virus titration by plaque assay on MDCK cell monolayers. Data represent the mean and n = 3–5 mice per group. * = p<0.01 # = p<0.05.

As there was substantial cross-reactivity *in vitro* (Fig. 2) for the D^b^NP_366_ and D^b^NPN3A-specific responses, it was important to determine whether memory T cells that cross-react for the D^b^NP_366_ and D^b^NPN3A epitopes are preferentially recalled by secondary infection with the heterologous virus. Mice that were primed with the PR8NPN3A and then challenged with either the wt HK or mutant HKNPN3A viruses showed equivalent recall of D^b^NPN3A^+^CD8^+^ T cells during the acute phase of the secondary response. This was detected by IFN-γ production (Fig 5A) and tetramer staining (data not shown) and is consistent with the TCR CDR3β analysis (Table 2). Conversely, when mice were firstly primed with wt PR8, then later infected i.n. with either the wt HK or mutant HK-NPN3A, the D^b^NP_366_^+^CD8^+^ T cells were differentially recalled indicating that (in the absence of primary selection from the naïve repertoire by the mutant epitope) only some of the D^b^NP_366_^+^CD8^+^ memory T cells that were expanded by heterologous challenge bind D^b^NPN3A (Fig. 5A). Interestingly, wt priming and challenge (1^0^ PR8->2^0^ X31) resulted in significantly higher CD8^+^ T cells numbers (Fig. 5B) than were found for any secondary CD8^+^ T cell responses after NPN3A priming (1^0^ PR8-NPN3A->2^0^ X31-NPN3A and 1^0^ PR8-NPN3A->2^0^ X31). These results lead to two main conclusions: (i) priming with the wt virus elicits CD8^+^ T cells that respond relatively well to a subsequent infection with cross-reactive variant (ie 1^0^ PR8->2^0^ X31-NPN3A = 1^0^ PR8-NPN3A->2^0^ X31-NPN3A); (ii) priming with the cross-reactive variant can be detrimental as the diminished primary response may limit the full expansion of CD8^+^ T cells that are able to respond to the subsequent wt infection (ie 1^0^ PR8-NPN3A->2^0^ X31 is lower than 1^0^ PR8->2^0^ X31) and skew the TCR usage. Thus, using NPN3A for either priming or the challenge gives an equally poor response.

To determine whether such decreased CD8^+^ responses elicited after heterologous challenge would affect influenza virus clearance, we performed experiments to examine the protective efficacy of cross-reactive CD8^+^ T cell repertoires. We performed prime-and-challenge studies in μMT mice lacking B cells to ensure that antibody responses did not mask any possible inhibitory effects of “suboptimal” TCRs on viral clearance. As suggested by CD8^+^ T cell data, assessment of lung viral titres showed delayed viral clearance on d6 after the secondary infection in case of heterologous prime-and-boost (PR8->X31-NPN3A and PR8-NPN3A->X31) compared to homologous infections (PR8->X31 or PR8-NPN3A->X31-NPN3A) (Fig. 5B). These results indicate that recall of “suboptimal” TCRs for a single T cell specificity can lead to delayed viral clearance, despite the presence of other influenza CD8^+^ T cell responses (D^b^PA_224_^+^CD8^+^, D^b^PB1_62_^+^CD8^+^, K^b^NS1_114_^+^CD8^+^, K^b^PB1_703_^+^CD8^+^).

**Figure 6:**
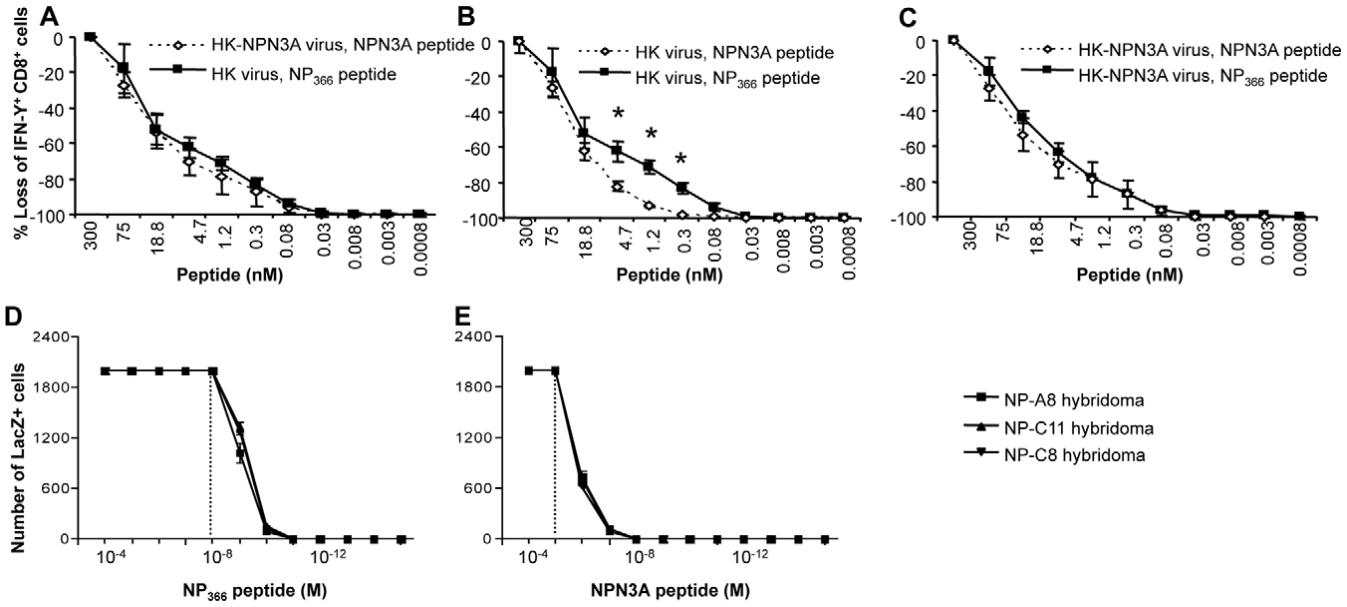
Decreased TCR functional avidity of D^b^NPN3A-primed T cells for D^b^NP_366_. (A–C) Responsiveness of CD8^+^ T cells to varying peptide concentrations was determined as a measure of functional TCR avidity for the cognate pMHCI complex. (A,B) D^b^NP_366_^+^ CD8^+^ and (A,C) D^b^NPN3A CD8^+^ T cells recovered from mice 2^o^-challenged with wt or NPN3A viruses were assessed for functional TCR avidity using the ICS assay. The enriched splenocytes analysed by IFN-γ ICS were stimulated *in vitro* with 4-fold dilutions of the NP_366_, or NPN3A peptide. Data represent mean±SD (n = 4), * = p<0.01. (D,E) The response profiles of monoclonal D^b^NP_366_-specific LacZ-inducible (22) T cell hybridomas (3 distinct lines all with the “public” SGGGNTGQL CDR3β sequence) to peptide-pulsed H-2D^b^ splenocytes presenting the (D) wt D^b^NP_366_ or (E) D^b^NPN3A epitopes were assessed in LacZ assay [22].

These patterns of complete, or partial, cross-stimulation following *in vivo* virus challenge were reflected in the results found for *in vitro* measurements of “functional avidity” (Fig 6). Pulsing immune spleen cells recovered directly *ex vivo* with graded doses of wt or mutant peptide in the ICS assay showed comparable levels of IFN-γ induction (Fig 6AC) in every situation but one, the exposure of wt-primed T cells to the NPN3A peptide (Fig 6B). Thus, while the immune repertoire selected by D^b^NP3A shows evidence of equivalent avidity following stimulation with the NPN3A or NP_366_ peptides, D^b^NP_366_ induces a response that is of higher avidity for the wt than the mutant epitope. The same effect was seen even more clearly when three (all expressing the SGGGNTGQL CDR3β) T cell hybridoma lines [37] specific for D^b^NP_366_ were stimulated with the two peptides (Fig 6DE). This result is in accord with findings from both the TCR repertoire analysis of cross-reactive clonotypes, assessed by tetramer binding (Table 2), and the response magnitudes determined following homologous and heterologous virus challenge (Fig 5). Thus, priming and recall of cross-reactive CD8^+^ T cells recognising mutant viral epitopes reflects functional (defined as responsiveness to a peptide) pMHC-TCR avidity.

**Figure 7:**
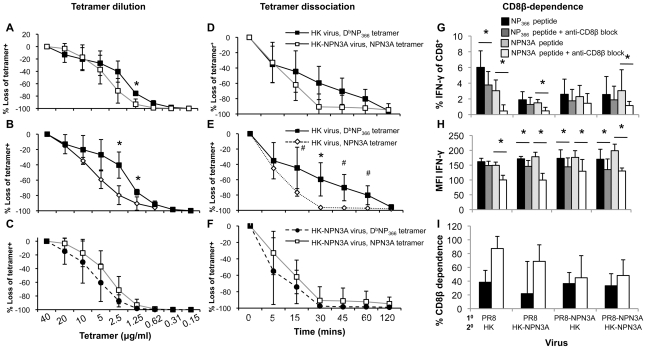
Decreased pMHC avidity and CD8β-independence for functional avidity of D^b^NPN3A-primed T cells for D^b^NP_366_. pMHC-TCR avidity was assessed by three measures: (A–C) the overall pMHC avidity (tetramer “on” and “off” rates) by tetramer dilution, (D–F) tetramer “off”-rate by tetramer dissociation and (G–I) CD8β-dependence by anti-CD8β blocking combined with ICS. Splenocytes were obtained from mice 2^o^-challenged with wt or NPN3A viruses. (A–C) D^b^NP_366_^+^ CD8^+^ and D^b^NPN3A CD8^+^ T cells were stained with 2-fold dilutions of PE-conjugated tetramers, followed by anti-CD8 staining. (D–F) Cells were stained with either the D^b^NP_336_ or D^b^NPN3A tetramers at the saturating concentration, then incubated at 37^o^C with a mAb to H2D^b^ to prevent rebinding of dissociated tetramer. The progressive diminution in tetramer staining was measured by flow cytometric analysis. (G–I) Lymphocytes were pre-cultured in the presence or absence of anti-CD8β antibody (53.5-8) (10 µg/ml). Cells were then stimulated for 5 h with peptide, IL-2 and GolgiStop also in the presence or absence of anti-CD8β antibody (5 µg/ml). Following stimulation, cells were analysed for CD8 and IFNγ expression. Shown is (G) the percentage of CD8^+^ T cells producing IFN-γ in the presence or absence of anti-CD8β blocking mAb, (H) mean fluorescence intensity (MFI) of IFN-γ staining, (I) the percentage of CD8^+^ cells dependent on anti-CD8β for IFN-γ production. Tetramer staining was performed in the presence of NaAz, then washed and incubated with anti-CD8 mAb. The progressive diminution in tetramer staining was measured. The Td50 value defining the time to 50% tetramer loss. Data represent mean±SD (n = 4–5 mice per group), *p<0.01; ^#^p<0.05.

To determine the pMHC-I avidity of the responding D^b^NP_366_^+^CD8^+^ and NPN3A^+^CD8^+^ T cells, we additionally performed tetramer dilution (Fig. 7A–C) and tetramer dissociation (Fig. 7D–E) assays. While tetramer dissociation assay measured the “off-rate” component of pMHC-I avidity, tetramer dilution technique assessed the overall pMHC-I avidity (both the “on” and “off” rates). Furthermore, we also assessed CD8β-dependence for functional activation (a measure of low avidity CD8^+^ T cells) of both wt D^b^NP_366_^+^CD8^+^ and the mutant NPN3A^+^CD8^+^ T cells by anti-CD8β mAb blocking, followed by IFN-γ ICS (Fig. 7G–I). Our data obtained from those three additional measures of pMHC-I avidity confirmed the results obtained by the peptide titration combined with ICS (functional avidity, Fig. 6) and further suggested significantly lower pMHC-I avidity of the wt D^b^NP_366_^+^CD8^+^ (generated by the wt HK infection) for NPN3A variant.

## Discussion

The P3N within the immunodominant influenza virus-specific D^b^NP_366_ epitope is a partially solvent exposed, and non-prominent for TCR binding, residue that is predominantly buried within the MHC cleft [21], [23]. The NPN3A mutation leads to both decreased recruitment of CD8^+^ T cells and a narrowed clonotype selection profile within Vβ8.3 and Vβ9 regions. Structurally, the mutation leads to a loss of a number of contacts between the NPN3A peptide and the MHC-I molecule, including a contact with the gate-keeper residue at position 155, and unaltered stability of the H-2D^b^-NPN3A complex. The fact that the NPN3A mutation affects contacts with the MHC-I at His155, known to play an important role in TCR-pMHC structures in general, is likely to indirectly compromise the TCR recognition. By loosing the bond with the Asn3, His155 may gain more flexibility and thus be inappropriately placed for the subsequent TCR ligation onto the NPN3A peptide. Alternatively, it is also possible that a small part of the solvent-exposed head group of the Asn3 residue in the wt NP_366_ peptide might, to some extent, be directly interacting with the TCR following ligation.

Surprisingly, despite the loss of several contacts between NPN3A peptide and H-2D^b^, the stability of the peptide-MHC-I complex remains constant for both NP_366_ and NPN3A. This suggests that the Asn3 as a secondary anchor does not play an important role in stabilizing the peptide within the MHC-I. The structural basis for the diminished recruitment of D^b^NPN3A-specific CD8^+^ T cells is thus likely to rest in the way that the partially-solvent exposed residue contacts MHC-I and modifies TCR ligation.

The emerging D^b^NPN3A^+^CD8^+^ T cell population was characterised by different Vα and Vβ preference, distinct CDR3β sequences and a lower overall TCR diversity in comparison to wt D^b^NP_366_^+^CD8^+^ T cells. These findings suggest that a very limited spectrum of CD8^+^ T cells can recognise the D^b^NPN3A mutant structure. Interestingly, a similar P3 substitution in the influenza virus D^b^PA_224_ epitope had no affect on D^b^PA_224_^+^CD8^+^ T cell recognition and recruited a diverse array of TCR clones comparable to the spectrum found for the wt response [21]. This suggests that the partially-exposed P3 residue plays a greater structural and/or TCR recognition role for the “featureless” D^b^NP_366_ than for the “featured” D^b^PA_224_ complex reflecting, in turn, the more limited spectrum of TCRs that bind D^b^NP_366_ [21]. Taken together, it appears that partially-exposed residues within viral peptides can provide important contacts with the MHC-I, which can in turn cause remote effects that modify antigenicity for the TCR-pMHC-I complex and impact on both TCR repertoire selection and the magnitude of CD8^+^ T cell responses.

Interestingly, there were no differences in function or phenotype characteristics for the D^b^NP_366_^+^CD8^+^ and NPN3A^+^CD8^+^ T cells, although those two CTL sets had a high proportion of different TCR clones. This is in accordance with previous studies showing that the simultaneous production of antiviral cytokines [29] and IL-7R [20] expression is antigen dose- rather than magnitude-related. Conversely, levels of the CD62L [38], [39], [40] activation marker differed for the D^b^NP_366_^+^CD8^+^ and NPN3A^+^CD8^+^ populations, indicating that response magnitude has some relationship to the activation status of CD8^+^ T cells [40], [41], [42] which may, perhaps, reflect the extent of CTL proliferation.

The TCR repertoires specific for D^b^NP_366_ and D^b^NPN3A appear to be quite distinct. The response overall for wt D^b^NP_366_^+^CD8^+^ T cells is characterised by conserved, “public” clonotypes that constitute the majority (83.5% in 1^0^ response and 92.3% in 2^0^ response) of the selected TCR repertoire [34]. These public clonotypes are not a prominent feature of the D^b^NPN3A^+^CD8^+^ set, being found only in 4/7 NPN3A-infected mice at very low frequency. Since we know that these TCR clonotypes are present in all B6 mice [34], the difference presumably reflects the lower TCR avidity for D^b^NPN3A, as indicated by the T cell hybridoma analysis where they were shown to require 1000 times more NPN3A than NP_366_ peptide for optimal stimulation. Since the public clonotypes cannot be efficiently recruited into the immune response by the mutated N3A virus, this could have created a “hole” in TCRs capable of recognising the mutated epitope, which subsequently can lead to a reduction in T cell immunogenicity [43]. However, though both the D^b^NP_366_^+^CD8^+^ and D^b^NPN3A^+^CD8^+^ T cell responses are characterised by quite distinct TCR repertoires, the majority are bound by both the D^b^NP_366_ and D^b^NPN3A tetramers and can be detected by stimulation with either the NP_366_ or the NPN3A peptides, suggesting that a clonal dissection of TCR clonotypes is needed to make a valid interpretation about the truly cross-reactive CD8^+^ T cell responses. These findings also raise questions concerning the true correlation between pHMC-I tetramer binding *in vitro* and the *in vivo* selection of a responding TCR repertoire.

Overall, the results indicate that a loss of a number of contacts between the NPN3A peptide and the MHC-I molecule and lower functional and structural pMHC-I avidity (for wt D^b^NP_366_) D^b^NPN3A selects a narrowed TCR repertoire of “best fit” TCRs from a spectrum of naïve clonotypes that, once activated, clonally expanded and engaged in an immune response, have sufficient avidity to be recalled by exposure to the wt D^b^NP_366_ epitope. Conversely, the “better” fit D^b^NP_366_ finds a sufficient spectrum of high avidity TCRs within that available naive repertoire and does not (likely because of clonal competition) select most of the TCR αβ pairs that interact optimally with D^b^NPN3A. Priming with the wt virus thus establishes memory for only a very limited secondary response to the mutant. Similar to our results, subtle variations within the anchor residue of H^b^ peptide/I-E^k^ also decreased peptide-MHC class II affinity and the activation of responding T cells [44].

Thinking about this in terms of possible vaccination strategies for use against rapidly changing viruses or tumor epitopes, it appears that priming with cross-reactive mutants that have characteristics comparable to NPN3A would be of no benefit (or even could be detrimental as evidenced by delayed viral clearance) as the same level of T cell immunity against such mutants can be elicited by exposure to the wt epitope. On the other hand, changes like the non-cross-reactive NP-M6A mutation [22] that induce a completely novel, high quality response might merit incorporation in an experimental vaccine or immunotherapy strategy. Overall, working out the topographical constraints that determine these differential response profiles would seem eminently worthwhile.

A further reason for defining the structural rules governing TCR cross-recognition is that similar effects have been found for different epitopes derived from unrelated viruses. Published studies provide evidence for cross-reactive CD8^+^ T cell responses between influenza A virus and Epstein-Barr virus (EBV) [45], influenza A virus and Hepatitis C virus (HCV) [46], [47] or lymphocytic choriomeningitis virus (LCMV) and Pichinde virus (PV) [48]. Such heterologous cross-reactive immunity can unintentionally skew TCR recruitment, result in a narrow TCR repertoire and subsequent viral escape [48] as well as influenza disease severity [45]. The topic of TCR cross-reactivity in CD8^+^ T cell responses clearly merits more attention.

## Materials and Methods

### Ethics statement

All animal experimentation was conducted following the Australian National Health and Medical Research Council Code of Practice for the Care and Use of Animals for Scientific Purposes guidelines for housing and care of laboratory animals and performed in accordance with Institutional regulations after pertinent review and approval by the University of Melbourne Animal Ethics Experimentation Committee in Melbourne.

### Mice and viral infection

C57BL/6J (B6, H2^b^) and μMT mice were bred and housed under specific pathogen free conditions at the Department of Microbiology and Immunology, University of Melbourne. For the generation of acute influenza responses mice were lightly anaesthetised by inhalation of methoxyflurane and infected intranasally (i.n.) with 1×10^4^ plaque forming units (p.f.u.) of HK-X31 (H3N2, X31) or modified HK-X31 (HK-NPN3A) influenza A viruses in 30µl of PBS. For recall responses mice were first primed intraperitoneally (i.p.) with 1.5×10^7^ p.f.u. of the serologically distinct PR8 (H1N1) or modified PR8 (PR8-NPN3A) influenza A viruses, in 500µl of PBS. Viruses share the same internal components for NP and PA from which CD8 epitopes are derived [49]. Virus stocks were grown in the allantoic cavity of 10 day old embryonated chicken eggs, from which the viral titre was determined by plaque assay on monolayers of Madin derby canine kidney (MDCK) cells.

### Generation and titration of recombinant influenza viruses

Recombinant influenza viruses with the single amino acid substitution (N3A) within the NP_366_ peptide, ASNENMETM, were generated using the eight-plasmid reverse genetics system [50]. The substitution was first incorporated by site directed mutagenesis using PCR primers encoding N3A_366_ peptide, ASAENMETM, and the opposite primer encoding NP. Recombinant PCR products encoding N3A_366_ were digested with B*sm*B1 and ligated into the alkaline phosphatase treated pHW2000 vector. Recombinant viruses (HK-NPN3A and PR8-NPN3A) were rescued following transfection of MDCK-293T cell co-culture with the eight plasmids encoding influenza segments. Viruses were then amplified in the allantoic cavity of 10-day old embryonated chicken eggs, and the viral titre determined by plaque assay of allantoic fluid infecting monolayers of MDCK cells. There was any evidence of altered fitness *in vivo* for the HKNPN3A virus, as the kinetics of virus growth and clearance following i.n. challenge of naïve B6 mice were found to be equivalent for the wt HK and mutant viruses (Fig S3A). Similarly, the levels of CTL activity found using target cells infected with HKNPN3A or the HK wt viruses were comparable, and the same was seen for peptide pulsed cells, suggesting that antigen presentation of NPN3A peptide remains constant (Fig S3 BC).

### Determination of viral titres

Lungs taken from mice after primary viral infection (Fig. S3A) or prime-and-boost approach using homologous (PR8->X31 and PR8-NPN3A->X31-NPN3A) or heterologous (PR8->X31-NPN3A and PR8-NPN3A->X31) strategy (Fig. 5B) were homogenised and the virus-containing supernatant above the cell debris was harvested and stored at −70°C. Titres of infectious virus in the lung supernatants were determined by plaque assay on monolayers of MDCK cells.

### Tissue sampling and cell preparation

Spleen and bronchoalveolar lavage (BAL) samples were recovered from mice at acute phases of the primary and secondary infections (d10 and d8 respectively), and the BAL samples were incubated on plastic petri-dishes for 1hr at 37^0^C to remove macrophages. The spleens were disrupted and enriched for CD8^+^ T cells using goat anti-mouse IgG and IgM antibodies (Jackson ImmunoResearch Labs, West Grove, PA, USA). For assessment of naïve precursor frequency of N3A_366_^+^CD8^+^ T cells, spleens and lymph nodes (inguinal, brachial, axillary, superficial cervical, and mesenteric) were collected from naïve mice and processed to single-cell suspensions.

### Tetramer and phenotypic staining of CD8^+^ T cells

Lymphocytes from the BAL and spleen were stained with tetramers conjugated to Strepavidin-APC or PE (Molecular Probes, Eugene, OR, USA) at optimal staining concentrations (10 µg/ml D^b^NP_366_, 40 µg/ml D^b^N3A_366_, and 10 µg/ml D^b^PA_224_ tetramers) for 1hr at room temperature. Cells were washed twice in FACS buffer, and stained with 1 µg/ml CD8-PerCP Cy5.5, 5 µg/ml CD62L-FITC and 5 µg/ml CD127-APC mAbs (BD Biosciences) for 30 mins on ice, washed twice and analysed by flow cytometry on the FACS Calibur (BD Immunocytometry) and analysed by CellQuest Pro software (BD Immunocytometry). We titrated all the batches of all the tetramers used in this study. We used tetramers at optimal concentrations (10–40µg/ml) based on both the percentage of epitope-specific CD8^+^ T cells and the mean fluorescence intensity (MFI) of tetramer staining. A Scatchard analysis [51] based on the tetramer dilution assay (Fig. 7 A–C) was also plotted (Fig. S4) and confirmed our observations from routine tetramer titrations that the D^b^NP_366_ tetramer displays slightly superior pMHC binding capacities over the NPN3A tetramer at a concentration <5µg/ml.

### Tetramer dilution and tetramer dissociation analyses

CD8^+^ T cells from spleen were stained with the D^b^NP_366_ and D^b^-NPN3A tetramers conjugated to Streptavidin-PE (Molecular Probes, Eugene, OR) for 60mins at room temperature. For a tetramer dilution assay, 2-fold dilutions of PE-conjugated tetramers were used at a range of concentrations (0.15–40µg/ml). For a tetramer dissociation assay, lymphocytes were stained at the optimal concentration of PE-conjugated tetramers as assessed by tetramer titration as determined by both the percentage of tetramer^+^CD8^+^ T cells and mean fluorescence intensity (MFI). Cells were washed twice in FACS buffer (10%BSA/0.02% NaAz in PBS), stained with a FITC-conjugated mAb to CD8α (BD Biosciences Pharmingen) for 30mins on ice, washed and analysed by flow cytometry. As a measure of pMHC avidity, splenic T cells were used in tetramer dissociation assay [29]. After staining with tetramer, T cells were washed and incubated in the presence of anti-H2D^b^ antibody at 5µg/ml at 37^0^C to prevent tetramer rebinding. Cells were removed at intervals, stained with the FITC-conjugated mAb to CD8α and analysed by flow cytometry. Loss of tetramer^+^CD8^+^ T cells at particular time-points was calculated in comparison to tetramer staining at t = 0mins.

### Peptide stimulation and intracellular cytokine staining

Enriched T cell populations from spleen and BAL were stimulated with one of the NP_366_, N3A_366_, PA_224_ or PB1_703_ peptides (AusPep) for 5 hrs at 37°C, 5% CO_2_ in the presence of 1µg/ml Golgi-Plug (BD Biosciences Pharmingen) and 10U/ml recombinant human IL-2 (Roche, Germany) (BD Biosciences). Cells were washed twice with FACS buffer, stained with CD8-PerCP Cy5.5 for 30 mins on ice, fixed, permeabilised and stained with anti-IFN-γ-FITC (5µg/ml), TNF-α-APC (2µg/ml), and IL-2-PE (2µg/ml) mAbs (Biolegend). Samples were acquired using flow cytometry, and the total cytokine production calculated by subtracting background fluorescence using no peptide controls. In selected experiments, lymphocytes were stimulated with varying concentrations of peptides, three-fold dilutions ranging from 300nM to 0.0008nM to determine the sensitivity specific peptides, defined as ‘functional avidity’ [52].

### TCR avidity for pMHC complex by CD8β-dependence

Splenocytes were obtained from mice sampled on d6 after secondary infection. Lymphocytes were pre-cultured in the presence or absence of anti-CD8β antibody (53.5–8) (10 µg/ml). Cells were then stimulated for 5 h with peptide, IL-2 and GolgiStop also in the presence or absence of anti-CD8β antibody (5 µg/ml). Following stimulation, cells were analysed for CD8 and IFNγ expression. Shown is the percentage of CD8^+^ cells producing IFN-γ after stimulation in the presence of anti-CD8β blocking mAb.

### Determination of N3A_366_^+^CD8^+^ T cell precursor frequency

Naïve N3A_366_-specific CD8^+^ T cells were identified as described [33], [53]. Briefly, processed lymph nodes and spleen samples were resuspended in 100 µl of Sorter buffer, 100 µl FcR block (24G2 and CD16 culture supernatant, 1% mouse and 1% rat serum) was added, and clumps of dead cells were discarded. Tetramers at optimal staining concentrations (D^b^N3A_366_-PE at 40µg/ml and D^b^NP_366_-PE at 10µg/ml) were added to the cell mix and incubated for 1 hour at room temperature in the dark. Cells were washed and resuspended in 400 µl buffer with 100 µl anti-PE microbeads (Miltenyi Biotec), and incubated at 4°C for 25 mins. Following two washes, cells were resuspended in 3 ml of buffer and cells that had bound the microbeads were purified on a magnetic LS column according to manufacturers instructions (Miltenyi Biotec). Cells eluted from the column were centrifuged (515×*g*, 6 min, 4°C), supernatant carefully aspirated to leave 90 µl buffer remaining, and 10 µl antibody cocktail was added for 30 min at 4°C. The antibody cocktail contained anti-CD8-APC Cy7 (Pharmingen, BD), anti-CD4-PE Cy7 (eBiosciences), anti-B220-FITC (Pharmingen, BD), anti-CD11b-FITC (eBiosciences), anti-CD11c-FITC (eBiosciences), anti-F4/80-FITC (eBiosciences), anti-CD62L-APC (Pharmingen, BD), and anti-CD3-PerCP Cy5.5 (Pharmingen, BD) mAbs. Cells were washed in 2 ml buffer, centrifuged (515×*g*, 6 min, 4°C), and supernatant aspirated leaving 100 µl. Resuspended cells were passed through 45 µm sieve, and data acquired by flow cytometry on the LSR II (Becton Dickinson) and analysed with FlowJo (Treestar Inc.) software. In selected experiments, naïve NPN3A^+^Vβ8.3^+^CD8^+^ T cells were single-cell sorted for TCRβ analysis. Experimental details of the single cell RT-PCR designed to amplify naive NPN3A^+^Vβ8.3^+^CD8^+^ T cells using 2 different sorting strategies are listed in Table S3.

### Hybridoma LacZ assay

LacZ-inducible T cell hybridomas specific for NP_366_ peptide [37], [54] were resuspended at 1×10^6^cell/mL and aliquots (100µl) and dispensed into 96-well flat-bottom plates together with 5×10^5^ naïve splenocytes (APCs). Cells were cultured in the presence of NP_366_ or NPN3A peptides at concentrations ranging from 10^−15^M to 10^−4^M for 18h at 37^0^C. The cells were then washed with PBS, fixed with 100µl of 2% formaldehyde/0.2% glutaraldehyde in PBS for 5min on ice, washed in PBS and incubated with 2.5mg of 5-bromo-4-chloro-3-indolyl β-D-galactoside (X-gal) for 16h at 37^0^C. The LacZ^+^ hybridomas were then counted using a light microscope, and the number of NP_366_-specific hybridomas was calculated by subtracting the “background” LacZ expression for cells cultured in the absence of the peptide.

### Isolation of single cell CD8^+^ T cells, RT-PCR and sequencing

Splenocytes were stained with 10 µg/ml D^b^NP_366_ or 40 µg/ml D^b^N3A_366_-PE tetramer in sort buffer (PBS with 0.1% BSA) for 60 mins at room temperature, washed and stained with 1 µg/ml anti-CD8-Allophycocyanin and 10 µg/ml of either anti-Vβ 8.3 or anti-Vβ9 for 30 mins on ice, washed twice with sort buffer. Single lymphocytes were isolated by sorting with a FACS Aria (BD Immunocytometry), into 80 wells of an empty 96 well twin-tec plate (Eppendorf). mRNA transcripts were reversed transcribed to cDNA, using a Sensiscript kit (Qiagen) according to manufacturers instructions, and the CDR3β region amplified by a nested hot start PCR using Vβ primers [34]. Positive PCR products were purified using Qiagen PCR purification kit and sequenced.

### Protein expression, purification, crystallisation and structure determination

H2-D^b^ and β2-microglobulin molecules were expressed in *Escherichia Coli* as inclusion bodies, refolded with the NP-N3A (ASAENMETM) peptide and purified as previously described [55], [56]. The H2D^b^NP-N3A complex crystals were obtained at 3mg/ml by the hanging-drop vapour diffusion technique at 20°C. Crystals were grown with a reservoir containing 0.1 M sodium citrate at pH 5.7, 28% PEG 3350 (w/v), 0.2 M lithium sulphate. The crystals belong to space group ***I222*** and the unit cell dimensions were consistent with one molecule per asymmetric units (Table S1).

The crystals were flash frozen to a temperature of 100K before data collection in-house with a Rigaku RU-200 rotating-anode X-ray generator. The data were processed and scaled with the XDS [57]. The crystal structure was solved using the molecular replacement method using the program Phaser [58] from the CCP4 suite of programs [59]. The search probe used to solve the structure was the structure of mouse MHC class I H2-D^b^ minus the peptide (Protein Data Bank accession number 3CPL) [22]. The progress of refinement was monitored by the R_free_ value with neither a sigma nor a low-resolution cutoff being applied to the data. The refinement protocol used includes several cycles of refinement with REFMAC [59] followed by manual model rebuilding with O program [60]. ‘Translation, liberation and screw-rotation’ displacement refinement was used to model anisotropic displacements of defined domains was used during the refinement process. The electron density around the NPN3A peptide was unambiguous, and all the side chains were built at full occupancy. Some mobile loops in the heavy chain of the H-2D^b^ molecule (residues 191–201; 220–228 and 247–254) have been removed from the final model due to missing electronic density. Final refinement statistics are summarized in Table I, the coordinates of the H2Db-NP-N3A complex have been deposited with the Protein Data Bank under accession numbers 3FTG.

### Thermostability measurements of recombinant class I complexes using circular dichroism (CD)

Circular Dichroism Spectra were measured on a Jasco 815 spectropolarimeter using a thermostatically controlled cuvette. A far-UV spectra was collected from 190nm to 250nm. The UV minimum was determined as 219 nm for H2Db-NP-N3A. The measurements for the thermal melting experiments was made at the minimum for H2Db-NP-N3A, at intervals of 0.1°C at a rate of 1°C/min from 20°C to 90°C. The Jasco Spectra Manager software was used to view and smooth the traces and then the GraphPad Prism software was used to plot Temperature versus % unfolded. The midpoint of thermal denaturation (Tm) for each protein was determined as the point at which 50% unfolding was achieved. The measurements were done in duplicate at two concentrations (4µM and 2.2µM) in a solution of 10mM Tris pH 8, 150mM NaCl.

### Protein Data Bank accession number

The atomic coordinates have been deposited in the Protein Data Bank, www.pdb.org (PDB ID code 3FTG).

## Supporting Information

Figure S1 Phenotypic and functional characteristics of NPN3A^+^CD8^+^ T cells. (A–D) Tetramer^+^ CD8^+^ T cells were characterized for (A, B) CD62L and (C, D) IL-7Rα expression following primary (A, C; d10) or secondary (B, D; d8) challenge. (E–H) The ICS assay was used to measure cytokine production following in vitro stimulation with the cognate peptide following primary (E, G) or secondary (F, H) challenge for TNF-α^+^ (E, F) and IL-2^+^ within the IFN-γ^+^ set (G, H). Data represent mean±SD from 5 mice per group, * = p<0.01.
(2.47 MB TIF)

Figure S2 TCR Vβ usage in primary and secondary NPN3A^+^ CD8^+^ and D^b^NP_366_^+^CD8^+^ T cell responses. Primary (A, C) or secondary (B, D) responses were generated by infection with either the (A, B) HK-NPN3A or (C, D) HK virus. Splenocytes were stained with the (A, B) D^b^NPN3A or (C, D) D^b^NP_366_ tetramer, anti-CD8 and anti-Vβ mAbs conjugated with FITC, then the tetramer^+^CD8^+^ cells were analysed for profiles of Vβ staining. Shown are results for (A–D) individual mice (n = 4). S: spleen.
(1.33 MB TIF)

Figure S3 N3A substitution within NP_366_ does not affect antigen presentation or the rate of viral clearance. Effects of N3A substitution within NP_366_ on (A) viral clearance and (B, C) antigen presentation was assessed. (A) Naïve mice were infected with either the wt HK or mutant HK-NPN3A virus. Lungs were sampled at days 3, 6, or 8 after infection and homogenized for virus titration by plaque assay on MDCK cell monolayers. Data represent the mean and n = 5 mice per group. (B, C) *ex vivo* ^51^Cr-mediated cytotoxicity was assessed after incubation of target (H2^b^) EL-4 target cells either (B) infected with HK or HK-NPN3A virus or (C) pulsed with 1µM NP_366_ or NPN3A peptides.
(1.21 MB TIF)

Figure S4 Scatchard analysis of TCR avidity for pMHC by tetramer dilution assay at the acute secondary time point (d8). Scatchard analysis of tetramer dissociation and correlation coefficient (R^2^) based on tetramer binding MFI (Holmberg K et al, J Immunol 171:2427, 2003) are shown. Memory mice primed with either (A, D) PR8 or (B, C) PR8-NPN3A viruses were challenged with either (A, D) HK or (B, C) mutant HK-NPN3A virus. Data represent mean of MFI/tetramer concentration (µg/ml) versus MFI of tetramer staining, from 4 mice per group.
(1.96 MB TIF)

Table S1 Summary of TCRβ and TCRα repertoire for D^b^NP_366_ and D^b^NPN3A Vβ8.3^+^ T cells following 1^0^ and 2^0^ infection with the wt or mutant NPN3A influenza virus
(0.05 MB DOC)

Table S2 Frequency of TCRβs in D^b^NPN3A^+^ Vβ9^+^CD8^+^ T cells after 1^0^ mutant HK-NPN3A infection detected with either the D^b^NP_366_^+^ or D^b^NPN3A^+^ tetramer
(0.05 MB DOC)
