# Supplementary figures and images for: Protective Efficacy of Cross-Reactive CD8+ T Cells Recognising Mutant Viral Epitopes Depends on Peptide-MHC-I Structural Interactions and T Cell Activation Threshold
Source: PLoS Pathog. 2010 Aug 12;6(8):e1001039. doi: 10.1371/journal.ppat.1001039 (PMC2920842; doi:10.1371/journal.ppat.1001039)

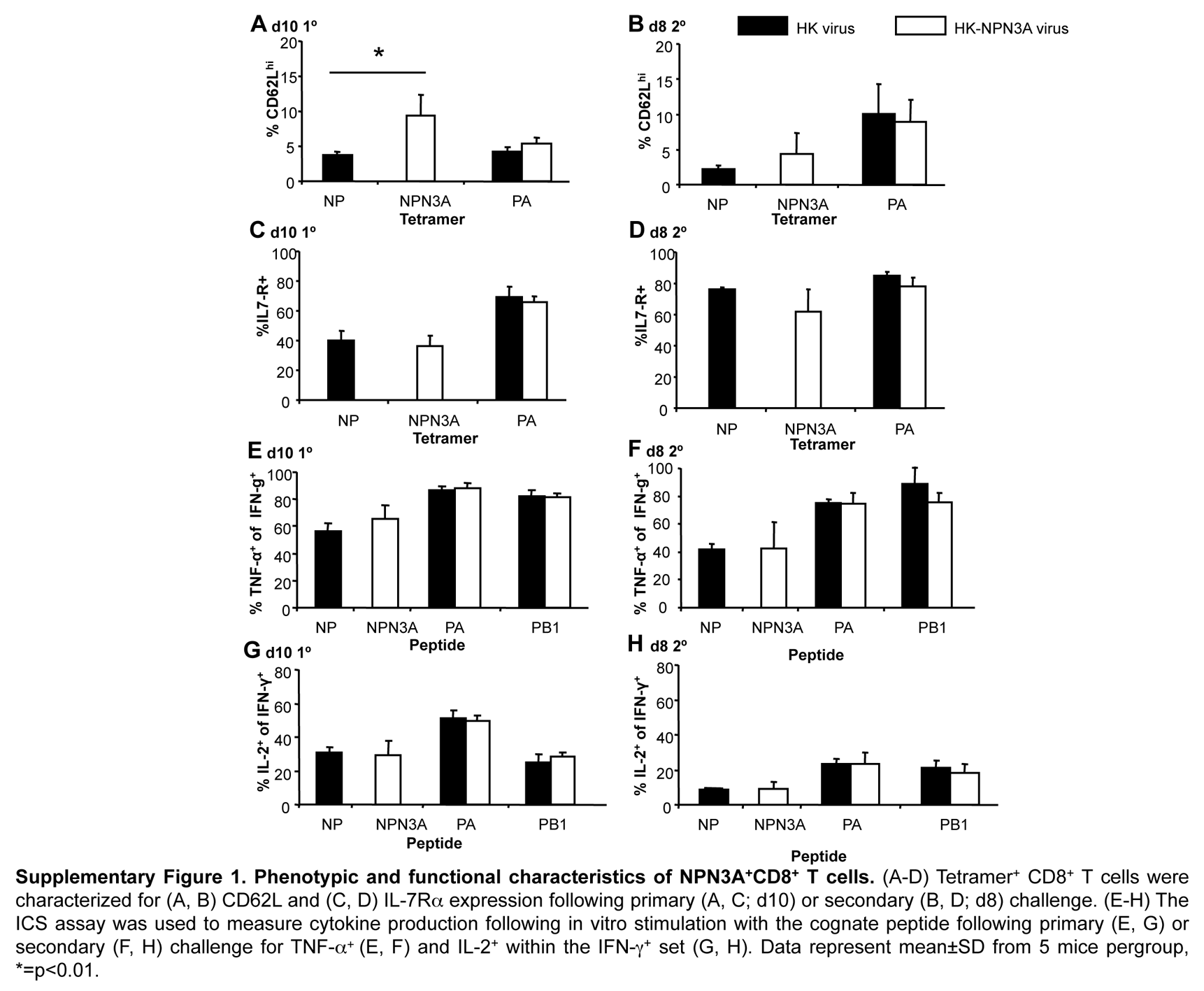

Supplement: Figure S1 — Phenotypic and functional characteristics of NPN3A+CD8+ T cells. (A–D) Tetramer+ CD8+ T cells were characterized for (A, B) CD62L and (C, D) IL-7Rα expression following primary (A, C; d10) or secondary (B, D; d8) challenge. (E–H) The ICS assay was used to measure cytokine production following in vitro stimulation with the cognate peptide following primary (E, G) or secondary (F, H) challenge for TNF-α+ (E, F) and IL-2+ within the IFN-γ+ set (G, H). Data represent mean±SD from 5 mice per group, * = p<0.01. (2.47 MB TIF) [file ppat.1001039.s001.tif]

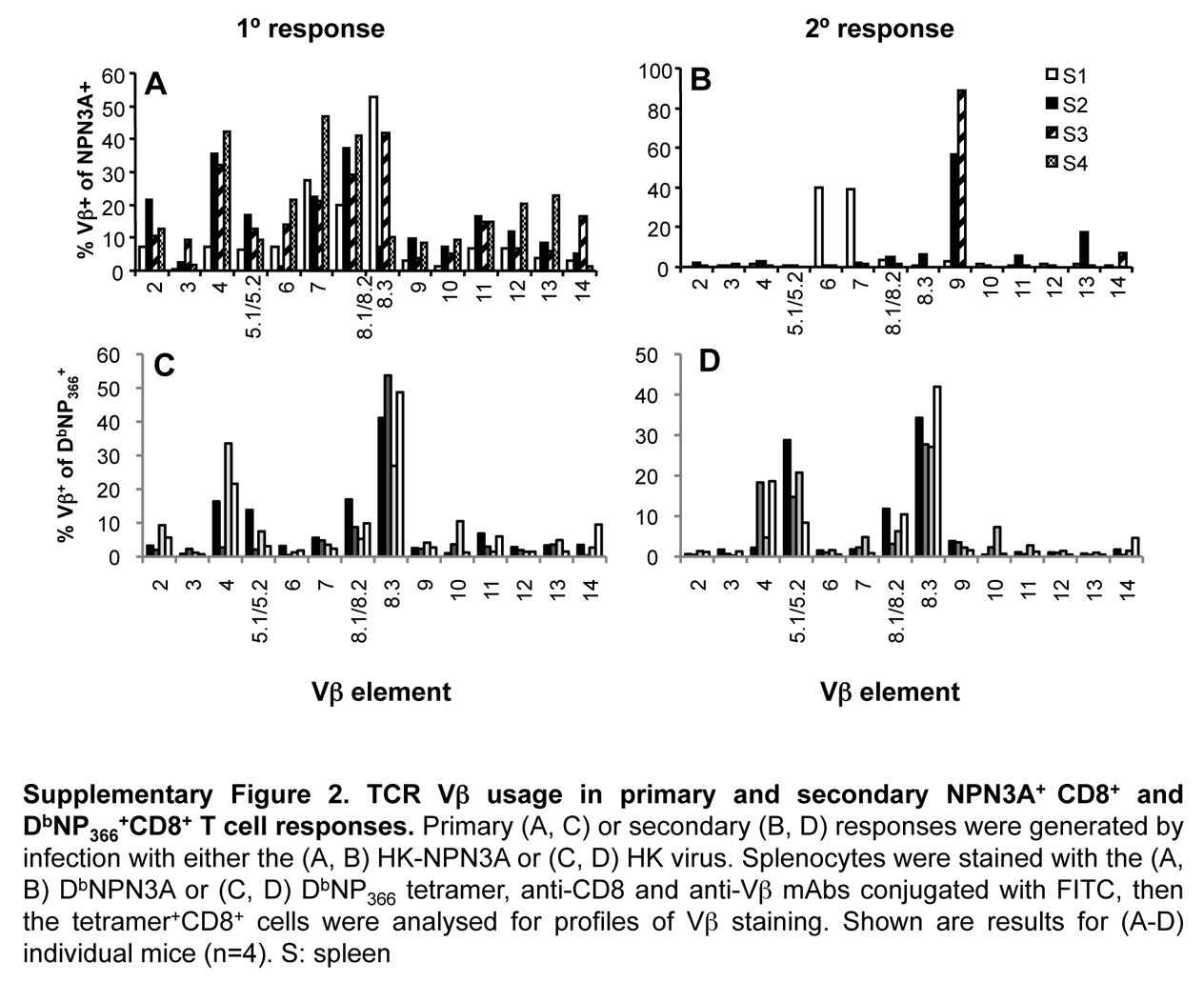

Supplement: Figure S2 — TCR Vβ usage in primary and secondary NPN3A+ CD8+ and DbNP366 +CD8+ T cell responses. Primary (A, C) or secondary (B, D) responses were generated by infection with either the (A, B) HK-NPN3A or (C, D) HK virus. Splenocytes were stained with the (A, B) DbNPN3A or (C, D) DbNP366 tetramer, anti-CD8 and anti-Vβ mAbs conjugated with FITC, then the tetramer+CD8+ cells were analysed for profiles of Vβ staining. Shown are results for (A–D) individual mice (n = 4). S: spleen. (1.33 MB TIF) [file ppat.1001039.s002.tif]

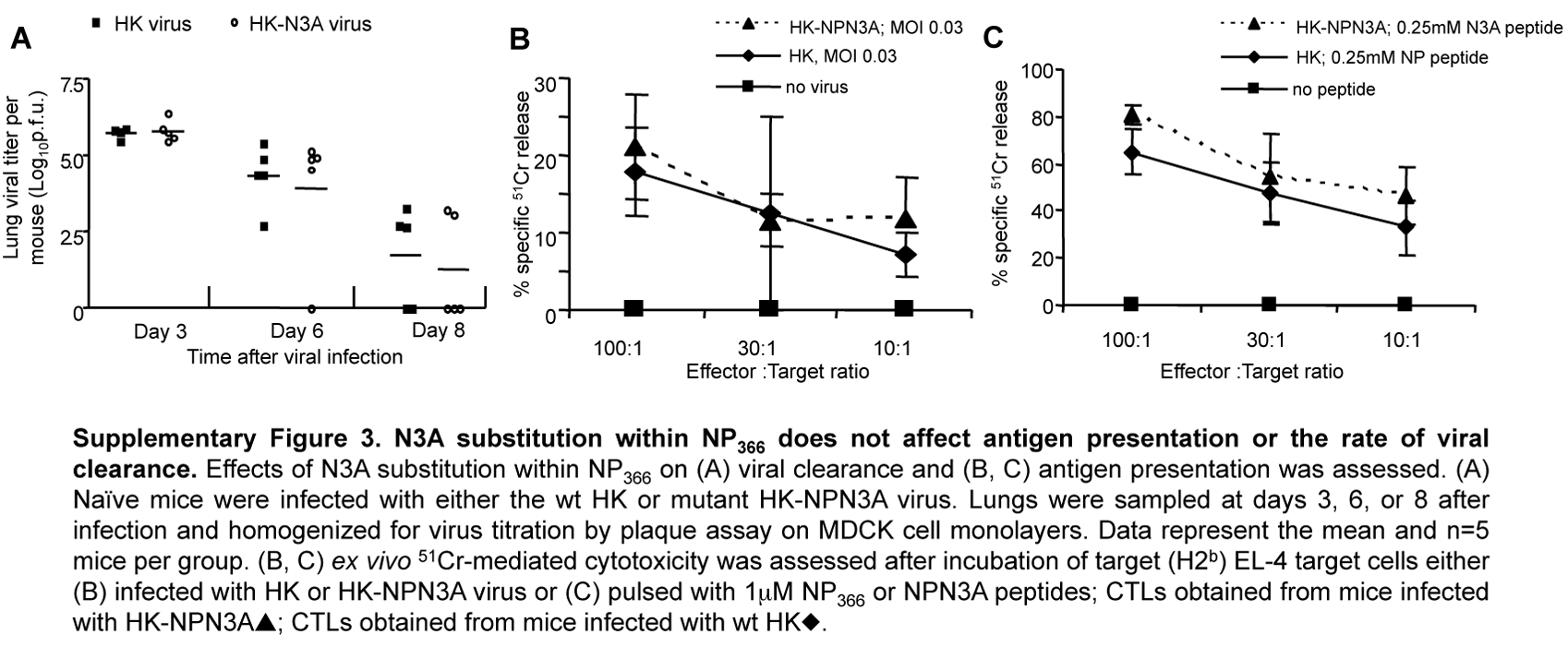

Supplement: Figure S3 — N3A substitution within NP366 does not affect antigen presentation or the rate of viral clearance. Effects of N3A substitution within NP366 on (A) viral clearance and (B, C) antigen presentation was assessed. (A) Naïve mice were infected with either the wt HK or mutant HK-NPN3A virus. Lungs were sampled at days 3, 6, or 8 after infection and homogenized for virus titration by plaque assay on MDCK cell monolayers. Data represent the mean and n = 5 mice per group. (B, C) ex vivo 51Cr-mediated cytotoxicity was assessed after incubation of target (H2b) EL-4 target cells either (B) infected with HK or HK-NPN3A virus or (C) pulsed with 1µM NP366 or NPN3A peptides. (1.21 MB TIF) [file ppat.1001039.s003.tif]

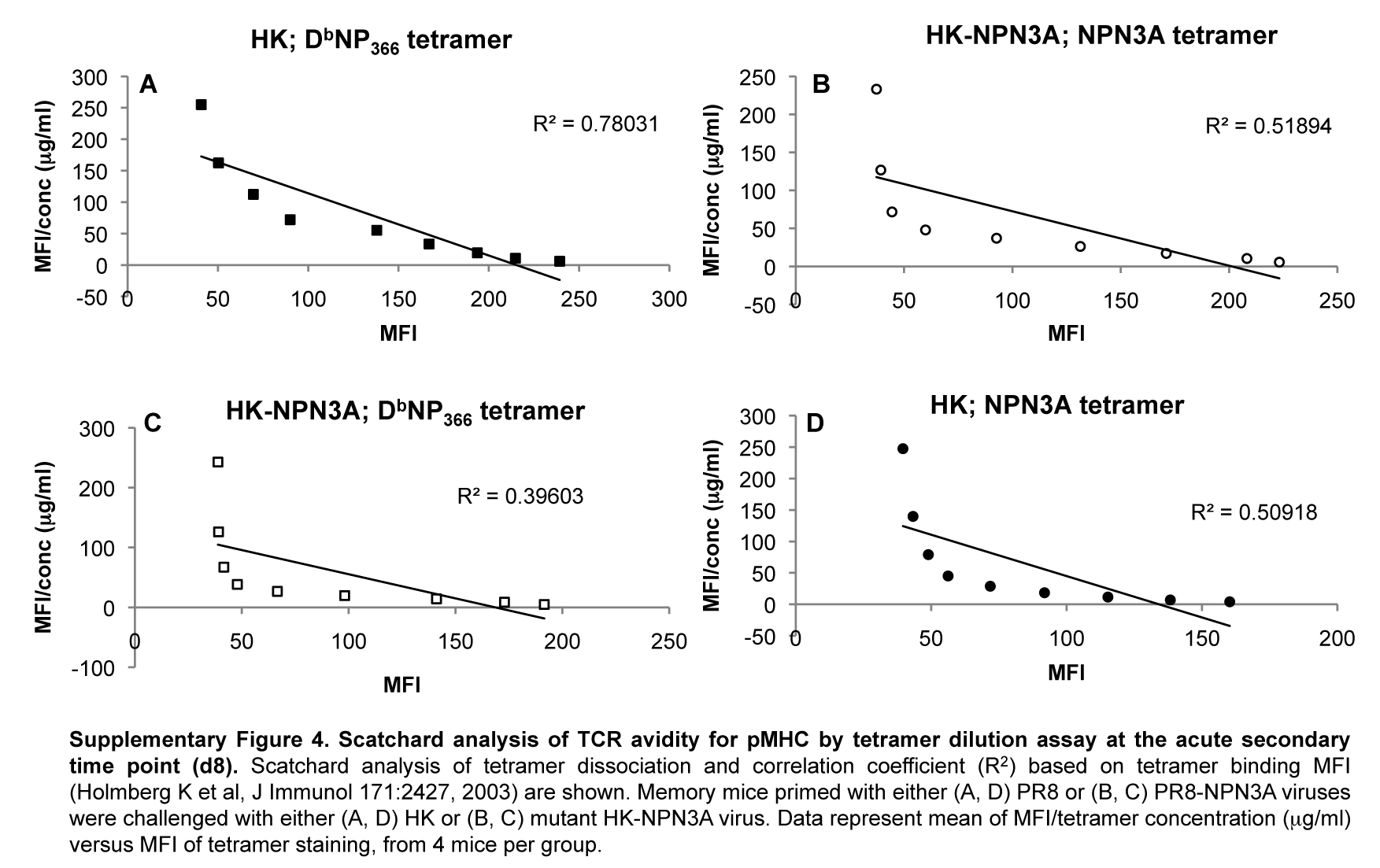

Supplement: Figure S4 — Scatchard analysis of TCR avidity for pMHC by tetramer dilution assay at the acute secondary time point (d8). Scatchard analysis of tetramer dissociation and correlation coefficient (R2) based on tetramer binding MFI (Holmberg K et al, J Immunol 171:2427, 2003) are shown. Memory mice primed with either (A, D) PR8 or (B, C) PR8-NPN3A viruses were challenged with either (A, D) HK or (B, C) mutant HK-NPN3A virus. Data represent mean of MFI/tetramer concentration (µg/ml) versus MFI of tetramer staining, from 4 mice per group. (1.96 MB TIF) [file ppat.1001039.s004.tif]
