# Supplementary material for: Protective Efficacy of Cross-Reactive CD8+ T Cells Recognising Mutant Viral Epitopes Depends on Peptide-MHC-I Structural Interactions and T Cell Activation Threshold
Source: PLoS Pathog. 2010 Aug 12;6(8):e1001039. doi: 10.1371/journal.ppat.1001039 (PMC2920842; doi:10.1371/journal.ppat.1001039)
Supplement: Table S1 — Summary of TCRβ and TCRα repertoire for DbNP366 and DbNPN3A Vβ8.3+ T cells following 10 and 20 infection with the wt or mutant NPN3A influenza virus (0.05 MB DOC) [file ppat.1001039.s005.doc]

**Table S1. Summary of TCRβ and TCRα repertoire for D^b^NP_366_ and D^b^NPN3A Vβ8.3^+^ T cells following 1^0^ and 2^0^** infection with the wt or mutant NPN3A influenza virus

|  | wt influenza virus infection | | | | Mutant NPN3A infection | | | |
| --- | --- | --- | --- | --- | --- | --- | --- | --- |
|  | 1^0^ response | | 2^0^ response | | 1^0^ response | | 2^0^ response | |
|  | D^b^NP_366_  tetramer | D^b^NPN3A  tetramer | D^b^NP_366_  tetramer | D^b^NPN3A  tetramer | D^b^NP_366_  tetramer | D^b^NPN3A  tetramer | D^b^NP_366_  tetramer | D^b^NPN3A  tetramer |
| **TCRβ repertoire** |  |  |  |  |  |  |  |  |
| Mice analysed | 3 | 3 | 3 | 3 | 2 | 2 | 5 | 5 |
| TCRs sequenced | 100 | 133 | 121 | 128 | 76 | 85 | 277 | 245 |
| Predominant* Jβ region | 2S2 | 2S2 | 2S2 | 2S2 | 1S1, 1S3 | 1S1, 1S3 | 1S1, 2S2, 2S4 | 1S1, 1S6, 2S2 |
| Predominant* CDR3β length (aa) | 9 | 9 | 9 | 9 | 9 | 8, 9 | 8, 9 | 8, 9 |
| Different clonotypes (aa) | 7 | 10 | 9 | 11 | 5 | 3 | 11 | 11 |
| Repeated clonotypes (aa) | 2 | 2 | 4 | 5 | 0 | 0 | 2 | 2 |
| Clonotypes per mouse (aa) | 4±1 | 4.3±0.6 | 5.3±0.6 | 6.3±2.1 | 2.5 | 1.5 | 2.6±1.5 | 3±1.4 |
| **TCRα repertoire** |  |  |  |  |  |  |  |  |
| Mice analysed | - | - | 3 | 3 | - | - | 3 | 3 |
| Predominant Vα region  (in all 3 mice) | - | - | 8, 17.3 | - | - | - | 8, 11 | 4, 5, 11 |
| Vα usage per mouse | - | - | 5.33±2.08 | 3.67±1.34 | - | - | 5.67±1.34 | 7±0 |

1^0^ responses were generated by i.n. HK or HK-NPN3A infection of mice; 2^0^ responses were generated by priming mice with i.p. PR8 or PR8-NPN3A viruses then challenging with i.n. HK or HK-NPN3A viruses respectively; D^b^NP_366_: complex of H2D^b^ and NP_366-374_ peptide; D^b^NPN3A: complex of H2D^b^ and NPN3A_366-374_ peptide.

*Predominant: ≥15%, ^#^Common: present in all mice sampled.
