# Supplementary material for: Protective Efficacy of Cross-Reactive CD8+ T Cells Recognising Mutant Viral Epitopes Depends on Peptide-MHC-I Structural Interactions and T Cell Activation Threshold
Source: PLoS Pathog. 2010 Aug 12;6(8):e1001039. doi: 10.1371/journal.ppat.1001039 (PMC2920842; doi:10.1371/journal.ppat.1001039)
Supplement: Table S2 — Frequency of TCRβs in DbNPN3A+ Vβ9+CD8+ T cells after 10 mutant HK-NPN3A infection detected with either the DbNP366 + or DbNPN3A+ tetramer (0.05 MB DOC) [file ppat.1001039.s006.doc]

**Table S2. Frequency of TCRβs in D^b^NPN3A^+^ Vβ9^+^CD8^+^ T cells after 1^0^ mutant HK-NPN3A infection detected with either the D^b^NP_366_^+^ or D^b^NPN3A^+^** tetramer.

| **HK-NPN3A infection** |  |  | **1^0^ response** | | | |
| --- | --- | --- | --- | --- | --- | --- |
|  |  |  | **M14** | | **M15** | |
| **CDR3β Vβ9** | **Jβ** | **aa** | **NP** | **N3A** | **NP** | **N3A** |
| SRDRNTL | 2S4 | 7 | 100 | 100 |  |  |
| SDRDRASDY | 1S2 | 9 |  |  | 97 | 100 |
| SGGKNTEV | 1S1 | 8 |  |  | 3 |  |
| **TOTAL sequences** |  |  | **39** | **48** | **39** | **48** |

M: individual mouse; NP: D^b^NP_366_ tetramer; N3A: D^b^NPN3A tetramer

1^0^ responses were generated by i.n. HK-NPN3A infection of mice;

D^b^NP_366_: complex of H2D^b^ and NP_366-374_ peptide; D^b^NPN3A: complex of H2D^b^ and NPN3A_366-374_ peptide.
